# Estuarine tidal range dynamics under rising sea levels

**DOI:** 10.1371/journal.pone.0257538

**Published:** 2021-09-20

**Authors:** Danial Khojasteh, Shengyang Chen, Stefan Felder, Valentin Heimhuber, William Glamore

**Affiliations:** Water Research Laboratory, School of Civil and Environmental Engineering, UNSW Sydney, Sydney, NSW, Australia; Universidade de Aveiro, PORTUGAL

## Abstract

How an estuary responds to sea level rise (SLR) is complex and depends on energy drivers (e.g., tides and river inflows), estuarine geometry (e.g., length and depth), intrinsic fluid properties (e.g., density), and bed/bank roughness. While changes to the tidal range under SLR can impact estuarine sediment transport, water quality, and vegetation communities, studies on the altered tidal range under SLR are often based on case studies with outcomes applicable to a specific site. As such, this study produced a large ensemble of estuarine hydrodynamic models (>1800) to provide a systematic understanding of how tidal range dynamics within different estuary types may change under various SLR and river inflow scenarios. The results indicated that SLR often amplifies the tidal range of different estuary types, except for short estuaries with a low tidal range at the mouth where SLR attenuates the tides. SLR alters the location of the points with minimum tidal range and overall tidal range patterns in an estuary. Variations in tidal range were more evident in converging estuaries, shallower systems, or in estuaries with strong river inflows. These findings provide an indication of how different estuary types may respond to estuaries and may assist estuarine managers and decision makers.

## Introduction

Estuarine environments provide a range of socio-economic and ecologic services, including primary production, water purification, recreational opportunities, navigational routes, and provision of nurseries for aquatic species [1–6]. However, a growing body of literature indicates that estuaries are likely to be vulnerable to sea level rise (SLR), as they are situated in low-elevation coastal areas, adjacent to the open ocean [7–10]. According to the Intergovernmental Panel on Climate Change (IPCC), global mean sea levels are estimated to rise between 0.28 m to 1.02 m by 2100, relative to the 1995–2014 average [11]. However, recent studies suggest that a global mean SLR of more than 2 m is possible by the end of the century due to uncertainties about the potential contributions of the Greenland and Antarctic ice sheets [12–14]. Therefore, the environmental and socio-economic impacts of SLR could be substantial [15, 16], particularly to the 630 million people who live on land below the projected upper range of high tide levels [17].

Estuarine tidal dynamics are complex and primarily controlled by energy drivers (i.e., tides, river inflows, waves, wind), geometry (e.g., length, width, depth, entrance condition, intertidal areas, slope), intrinsic fluid properties (e.g., density, viscosity), and other elements (e.g., roughness, protective structures) [10, 18, 19]. Interactions between these mechanisms typically determine the tidal dynamics (e.g., tidal range, prism, current) of an estuary and influence the processes within the estuarine system [20, 21]. As such, an accurate prediction of the tidal dynamics is required to assist managers and policy makers in developing long-term estuarine management plans, particularly under accelerating SLR [18, 20, 22, 23].

Analysing the tidal range (i.e., the difference between the high and low tide) is useful in gaining insights into how estuaries will respond to future SLR, since the tidal range is closely linked with mixing, circulation, sediment transport, water quality, and vegetation/ecosystem communities [24–27]. For instance, in a frictionless and reflectionless system, tidal range can be amplified with decreasing width and depth as the energy flux remains constant (for details, see [28]). Further, in converging estuaries, the concentration of energy as it is funnelled from the open ocean into the estuary can increase the tidal range, such as in the Severn River estuary and Bristol Channel [29]. On the other hand, strong river inflows (*Q*) are an additional source of energy that can attenuate the tidal range in upstream reaches [30, 31], modifying the direction and strength of flood/ebb currents [32].

Changes in the estuarine tidal range can also affect the structure of tidal currents and thereby, sediment transport dynamics [33, 34]. The process of sediment erosion, suspension, and deposition, as well as the hydroperiod (i.e., depth, duration, and frequency of tidal inundation), influences the establishment of vegetation communities and their distribution/extent within an estuary [26, 35–37]. As such, SLR-induced variations in tidal range dynamics are likely to pose a significant challenge within many estuaries, requiring further attention.

Estuarine tidal range responses to SLR are complex and site specific (depending on energy drivers, shape, and roughness). However, most analytical and semi-analytical studies have primarily focused on tidal wave physics, saltwater intrusion, and the effect of channel dredging (e.g., [28, 38–40]), without a focus on SLR. Where sufficient datasets exist (e.g., accurate bathymetry, time series of river discharge, etc), hydrodynamic modelling studies have indicated that SLR induces spatially disparate changes to the tidal range [21, 41, 42]. For instance, tidal range is predicted to: increase under SLR within the Mobile, Perdido, and Choctawhatchee Bays, USA [43, 44], Chesapeake Bay, USA [41, 45, 46], Delaware Bay, USA (if overland flooding is prevented) [42, 45], and Pearl River estuary, China [47]; decrease within the Choptank River estuary, USA [41], Patapsco River estuary, USA [41], and Delaware Bay, USA (if overland flooding is allowed) [42]; and negligibly change within St. Andrew and Pensacola Bays, USA [44], and the Tamar River estuary, Australia [48]. Nonlinear responses of estuarine tidal range dynamics to SLR were also highlighted in [41], through the use of 40 idealised simulated cases.

To date, existing studies have considered compound flooding (interaction of tides, river inflows, storm surges, and SLR) [49, 50], or a systematic analysis of the influence of river inflows on tides [51]. However, a parameter space study to investigate the combined influence of river inflows and SLR on tides is still absent. Further, current knowledge is mainly based on case studies that provide insights into a specific estuary in a certain time period and cannot be transferred more broadly. As such, the primary motivation of this study is to expand on previous analytical, semi-analytical, and idealised studies and to provide systematic insights into tidal range dynamics within various estuary types with different boundary conditions and different SLR scenarios. Throughout the paper, the potential applications, and limitations of the applied method, as well as its implications for future estuary research, are discussed. Directions for future research are also presented.

In all, this study aims to answer the following questions:

1. How will SLR influence the tidal range of various estuary types?
2. What are the key parameters controlling estuarine tidal range responses to SLR?
3. How do varying river inflows influence estuarine tidal range dynamics under SLR?
4. Which estuary types are more susceptible to SLR-induced tidal range amplification?

## Methodology

### Numerical experiments

**Fig 1:**
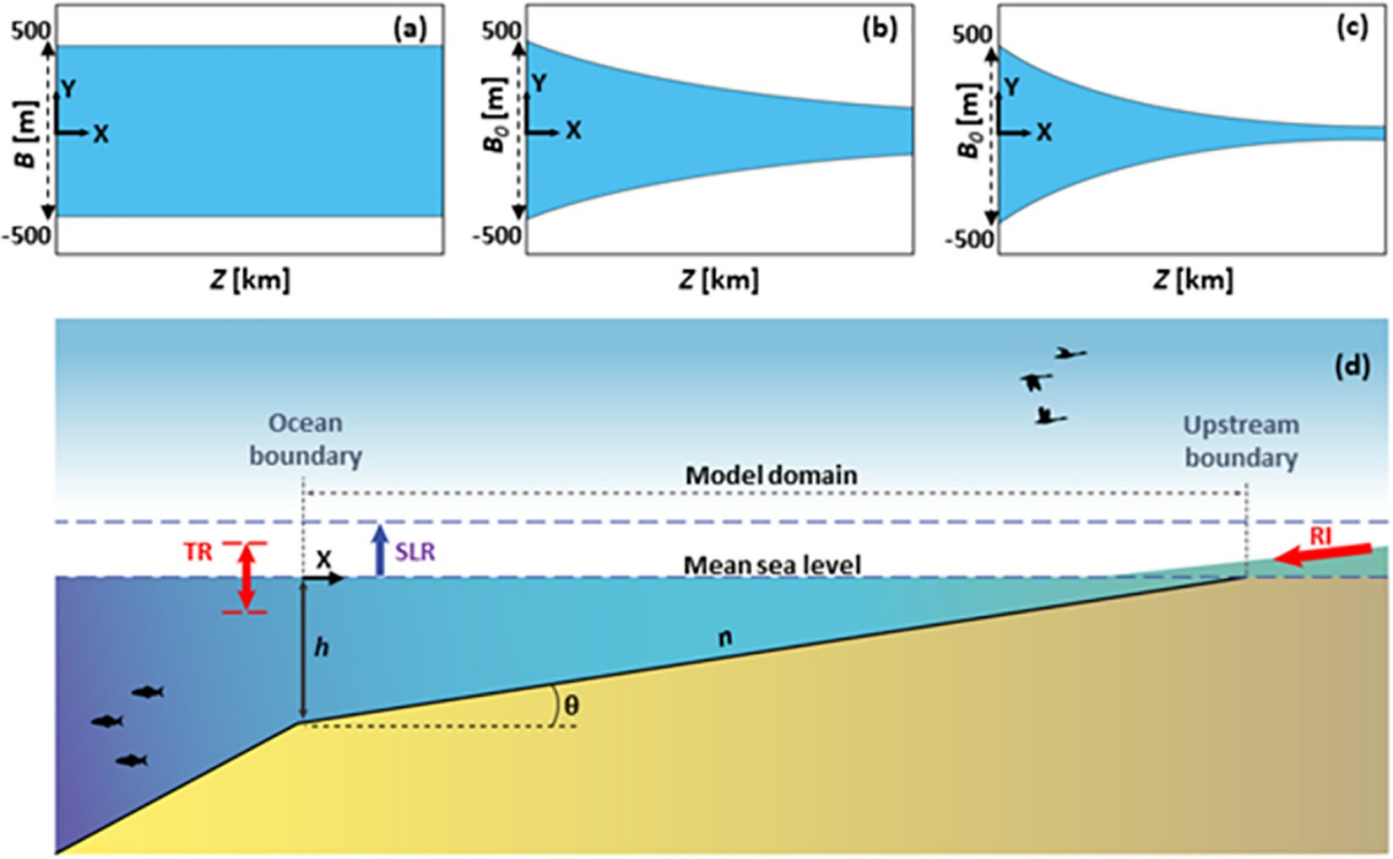
Estuarine geometries considered in this study; top view of (a) prismatic; (b) converging with *L*_*c*_ = 160 km; and (c) converging with *L*_*c*_ = 80 km estuaries. Panel (d) shows a side view of these geometries with the applied boundary conditions.

In this study, 1836 estuarine hydrodynamic simulations have been carried out to examine tidal range dynamics of different estuary types to SLR. Three generalised geometries have been selected including prismatic, weakly converging, and moderately converging estuaries, as they widely represent many real-world estuaries [28, 39, 41, 52–54]. Fig 1 schematically depicts the estuaries considered in this study together with definitions of the coordinate system and key parameters. Prismatic estuaries (e.g., Rotterdam Waterway) are mainly human-made and the banks of the estuary are made parallel through dredging and riverbank stabilisation [39] (Fig 1A). The width of many converging estuaries (e.g., Scheldt estuary) can be defined as an exponential function of distance from the mouth along the estuary axis *B*(*x*) = *B*_0_exp (−*x*/*L*_*c*_), where *x* is distance from the mouth, *B*_*0*_ is the width at the estuary mouth, and *L*_*c*_ is the width convergence length [39] (Fig 1B and 1C). Two values of *L*_*c*_ = 160 and 80 km were used to represent a wide range of weakly and moderately converging estuaries.

Several parameters have been varied throughout the simulations including tidal range at the mouth (*TR*_*0*_), estuary length (*Z*), estuary depth (*h*), Manning’s roughness (*n*), bed slope (θ), river inflow (*Q*), and SLR scenarios. All tested parameters and their values are summarised in Table 1. Tides at the mouth were assumed as a sinusoidal semi-diurnal tide M2 with a period *T* = 12.42 hours, as these tidal dynamics dominate in many locations worldwide, and hence, the present study potentially applies to semi-diurnal estuaries. Different tidal ranges at the mouth were examined to mimic micro-, meso-, and macro-tidal estuaries, hereafter called low (*TR*_*0*_ = 0.5 m), medium (*TR*_*0*_ = 1 m), and high (*TR*_*0*_ = 4 m) tidal ranges, respectively. The tides were applied directly at the ocean/estuary boundary (Fig 1D) as sensitivity tests indicated that a continental shelf domain has negligible influences on the tidal dynamics of the idealised cases considered in this study. For each case, three simulations were performed including a base case (without SLR), as well as SLR of 1 m and 2 m to cover SLR projections. The base cases yield a respective tidal prism (*TP*), here defined as the volume of water entering the estuary over a flood tide cycle. For cases with upstream river discharges, a constant river inflow with a desired percentage of tidal prism (*Q*/*TP* ratio) was then applied at the head (see Fig 1D). Four different ranges of *Q*/*TP* were considered to highlight variability of estuarine tidal range during no river discharge, as well as during low, medium, and high river discharge conditions. It is worth mentioning that both *TP* and *Q* were expressed as rates of flow in m^3^/s. Three different estuary lengths were considered including *Z* = 40, 80, and 160 km, hereafter named as short, moderate, and long estuaries, respectively. Three different Manning’s *n* were tested to highlight the importance of bank and bed roughness. The formulation and values of *n* were taken from [55] comprising low (*n* = 0.015 s/m^1/3^), moderate (*n* = 0.03 s/m^1/3^), and high (*n* = 0.09 s/m^1/3^) friction. It is worth noting that *n* = 0.09 s/m^1/3^ may rarely be observed in real-world conditions (e.g., systems with very thick Rhizophera mangroves) but is considered here to represent the upper limit of estuarine roughness. To check the effect of bed slope on tidal range dynamics, a flat bed (θ = 0°), and three further constant bed slopes (applied so that the water depth reduced linearly from the initial value (*h* = 5 m) at the mouth to zero at the head of the base cases) were examined (see Fig 1D and Table 1).

**Table 1:** Parameters and their values considered in this study to align with reported values of real-world estuaries.

Parameter	Value	Reference
Estuary length (*Z*) [km]	40, 80, 160	[41]
Estuary depth (*h*) [m]	5, 10	[39, 56]
Estuary width (*B*) [m]	1000	[57]
Tidal period (*T*) [hour]	12.42	[54]
Tidal range at the mouth (*TR*_*0*_) [m]	0.5, 1, 4	[21]
Manning’s coefficient (*n*) [s/m^1/3^]	0.015, 0.03, 0.09	[55]
River inflow/Tidal prism (*Q*/*TP*) [%]	0, 1, 5, 10	[8]
Bed slope (θ) [°]	0, 0.002, 0.004, 0.007	[58]
Sea level rise (SLR) [m]	0, 1, 2	[11, 59]

### Model description and validation

Numerical modelling of the idealised estuaries was undertaken using the RMA-2 modelling suite [60]. RMA-2 solves the depth-averaged shallow water wave equations and is suitable for the simulation of flow in well-mixed water bodies such as rivers and estuaries [61]. RMA-2 uses the principles of conservation of mass and momentum and represents typical processes of bed and bank friction as well as turbulence characteristics by using eddy viscosity coefficients. It calculates the Galerkin finite element solution of the shallow water equations. The two-dimensional system can be represented by triangular and quadrilateral elements with quadratic distributions of velocity and linear distributions of water depth. The stability of the model is not limited by the Courant condition as it is programmed with a Crank Nicholson implicit time integration scheme for transient conditions [7]. Further information regarding the RMA-2 modelling package is available in [60, 62–65].

After conducting a grid independency study, quadrilateral elements with a mesh resolution of 100 m were selected to discretise/grid all present estuaries, while ensuring optimum computational efficiency and adequately representing the flow characteristics. Each model was run for a period of 30 days with a time step of 15 minutes. Water levels and flow velocities were then computed and saved for every node over the simulation time. To avoid instabilities in the initialisation process, the water level and flow velocity data was discarded for the first 10 days of each run. The predictive accuracy of the model was previously studied and verified by [66] through a comparison of the tidal ranges of prismatic and converging estuaries against analytical solutions [28]. The root mean-squared error (RMSE) and the correlation coefficient (R^2^) indicated that the present idealised framework is able to reproduce the tidal dynamics predicted by the analytical method with acceptable accuracy (for details, see [66]). For short estuaries (*Z* = 40 km), RMSE and R^2^ were 2.5–3.8 cm and 0.99, respectively. These parameters were 3.1–8.7 cm and 0.95–0.97, as well as 3.2–3.6 cm and 0.92–0.99 for moderate (*Z* = 80 km) and long (*Z* = 160 km) estuaries, respectively.

### Parameter space analysis

As suggested by [38, 56], estuaries can be classified as weakly, moderately, and strongly converging as well as weakly, moderately, and strongly dissipative based on parameters *K*, *S*, and *R*:

$$K=\frac{U^{*}}{\varepsilon \omega^{*}L_{c}}$$ (1)

$$S=\frac{\omega^{*}L^{*}F^{2}}{\varepsilon U^{*}}$$ (2)

$$R=\frac{L^{*}F^{2}}{\varepsilon h{C^{*}}^{2}}$$ (3)

Where ω* is the angular frequency of the tidal wave, *ε* is the ratio of tidal amplitude over average depth, and *F* is the Froude number. U*, L*, and C* are characteristic amplitude of tidal velocity, horizontal length scale describing spatial variations in flow characteristics, and the flow conductance, respectively, defined as [56]:

$$U^{*}=\sqrt[{3}]{\frac{{ga}^{2}{C^{*}}^{2}}{T}}$$ (4)

$$L^{*}=\sqrt{\frac{gTh^{2}{C^{*}}^{2}}{U^{*}}}$$ (5)

$$C^{*}=\frac{h^{1/6}}{n\sqrt{g}}$$ (6)

Where *a* is the tidal amplitude (half of tidal range). Using dimensionless parameters of *K* and *R*/*S*, estuaries can be generally classified as weakly converging (WC) 0<*K*<0.5, moderately converging (MC) 0.5≤*K*<1, and strongly converging (SC) *K*≥1, as well as weakly dissipative (WD) 0<*R*/*S*<0.5, moderately dissipative (MD) 0.5≤*R*/*S*<2, and strongly dissipative (SD) *R*/*S*≥2 (for details, see [56]).

Based on the range of variables tested in this study, prismatic (*K*≅0), weakly, and moderately converging (0.02<*K*<0.7) as well as weakly, moderately, and strongly dissipative (0.14<*R*/*S*<17) estuaries were considered. This parameter space can cover a wide range of real-world estuary types as presented in Table 2, where it is indicated that the model simulations can be applied to 68% of these selected sites taken from existing analytical and semi-analytical studies (34 out of 50 sites). This is important as the relative strength of inertia and convergence versus dissipation (friction) could characterise the ebb or flood dominance. For weakly dissipative estuaries, the tidal wave propagation is weakly nonlinear, and increasing channel convergence intensifies the tidal wave distortion, resulting in ebb dominance [28, 56]. In contrast, tidal wave propagation is a nonlinear phenomenon in strongly dissipative estuaries, leading to substantial distortion of tidal wave and flood dominance [28, 56].

**Table 2:** Geometric and tidal properties of selected real-world estuaries extracted from existing analytical and semi-analytical studies along with measured dimensionless parameters defining estuarine classification. The values of *a*, *h*, *L*_*c*_, *T*, and *n* are taken from the corresponding references, and U*, L*, C*, *K*, and *R*/*S* are calculated using Eqs (1–6).

Estuary	*a* [m]	*h* [m]	*L*_*c*_ [km]	*T* [hour]	*n* [s/m^1/3^]	U* [m/s]	L* [km]	C* [–]	*K* [–]	*R*/*S* [–]	Reference
Delaware	0.64	5.8	40	12.5	0.020	0.34	140.5	21.4	0.56	0.93	[45]
Chesapeake	0.38	7	175	12.5	0.025	0.21	177.5	17.7	0.16	0.70	[45]
Elbe	1.5	16.5	50	12.5	0.022	0.64	317.1	23.2	1.01	0.42	[67, 68]
Scheldt	2.1	10	25	12.5	0.022	0.76	162.5	21.3	1.03	1.20	[67, 68]
Hau	1.4	7.5	51	12.4	0.022	0.56	134.4	20.3	0.42	1.29	[67, 69]
Tien	1.05	8.2	56	12.4	0.022	0.47	163.4	20.6	0.46	0.96	[67, 69]
Bristol Channel	2.6	45	65	12.4	0.030	0.84	651.1	20.1	1.59	0.33	[56, 70]
Columbia	1	10	25	12.4	0.026	0.41	185.2	18.1	1.2	0.91	[56, 70]
Conwy	2.4	3	6.3	12.5	0.020 ^‡^	0.77	43.5	19.2	1.10	5.02	[56]
Fraser	1.5	9	215	12.4	0.032	0.47	125.3	14.4	0.93	1.78	[56, 70]
Outer Bay of Fundy	2.1	60	230	12.4	0.030	0.75	962.5	21.1	0.67	0.20	[56, 70]
Gironde	2.3	10	44	12.4	0.026	0.72	140.3	18.0	0.51	1.60	[56, 70]
Hooghly	2.1	5.9	72	12	0.020	0.77	93.8	21.5	0.21	1.96	[56, 71]
Ord	2.5	4	15	12.4	0.020	0.82	58.8	20.1	0.62	3.61	[56, 70]
Irrawaddy	1	12	35	12	0.014	0.65	335.2	34.5	1.52	0.31	[56, 72]
Potomac	0.65	6	54	12.4	0.018	0.38	154.9	23.9	0.46	0.78	[56, 70]
Severn	3	15	41	12.4	0.025	0.93	206.8	20.1	0.80	1.09	[56, 70]
St. Lawrence	2.5	7	183	12.4	0.023	0.80	99.6	19.2	0.87	2.19	[56, 70]
Thames	2	8.5	25	12.3	0.032	0.56	106.3	14.3	0.68	2.30	[56, 70]
Tamar	2.6	2.9	21	12.5	0.020 ^‡^	0.81	40.8	19.1	0.31	5.52	[56]
Tees	1.5	7.5	5.5	12	0.028	0.51	109.4	15.9	3.20	1.82	[56, 70]
Gambia	0.6	8.7	56	12.4 ^‡^	0.024	0.31	198.4	19.1	0.56	0.69	[70, 73]
Pungwe	3	4.3	20	12.3	0.032	0.69	43.5	12.7	0.35	6.95	[70, 73]
Tha Chin	1.2	5.5	80	12.3	0.020	0.52	106.3	21.2	0.21	1.49	[70, 73]
Limpopo	0.55	7.1	200	12.3	0.023	0.29	166.7	19.2	0.13	0.78	[70, 73]
Maputo	1.75	3.9	16	12.3	0.014	0.82	81.1	28.6	0.81	1.81	[74]
Incomati	0.5	4	42	12.3	0.020	0.28	99.9	20.1	0.38	1.23	[70, 73]
Guadalquivir	0.95	7.1	65	12.42	0.022	0.43	144.1	20.1	0.35	1.07	[75]
Mae Klong	1.05	5.2	300	12.3	0.025	0.41	89.9	16.8	0.05	1.97	[39, 76]
Sinnamary	1.45	3.5	21	12.3	0.020	0.57	62.3	19.8	0.47	2.84	[74]
Corantijn	1.1	6.8	60	12.3	0.025	0.44	119.3	17.6	0.32	1.46	[74]
Humber	3	12	25	12.5	0.020^‡^	1.05	188.3	24.2	1.20	1.07	[68]
Weser	2	13	45	12.5	0.026	0.68	197.8	18.8	0.7	1.05	[68]
Loire	2.75	8	53	12.4	0.020^‡^	0.95	122.9	22.6	0.37	1.65	[77]
Vam (Van) Co	1.3	7	21	12.4^‡^	0.030	0.43	103.8	14.7	0.79	2.02	[77, 78]
Bernam	1.3	5.2	16.7	12.3	0.014	0.70	12.3	30.0	1.18	1.05	[79]
Selangor	1.6	3.6	13.4	12.3	0.025	0.52	51.95	15.8	0.62	4.08	[79]
Muar	1	7.9	31	12.3	0.022	0.45	158.4	20.5	0.81	0.96	[79]
Kurau	1	5.7	28	12.3	0.033	0.33	84.1	12.9	0.48	2.46	[79]
Perak	1.25	6.4	21	12.3	0.015	0.66	153.3	29.0	1.14	0.87	[79]
Endau	1	7.1	36	12.3	0.017	0.53	167.1	26.0	0.74	0.79	[79]
Sebou	1.1	5	50	12.45	0.020	0.49	99.2	20.9	0.32	1.59	[80]
Loukkos	1.55	4.6	17	12.42	0.025	0.52	69.4	16.5	0.65	2.98	[80]
Edisto	1.15	4	23	12.3	0.033	0.35	54.2	12.2	0.37	4.20	[74]
Tejo	1.8	5	13	12	0.018	0.73	88.1	23.2	1.08	1.88	[81]
Pangani	2.1	3.2	15	12	0.024	0.64	42.1	16.1	0.45	5.30	[81]
Linggi	1	3.2	13	12	0.033	0.32	43.6	11.7	0.53	4.91	[81]
Rompin	1.25	6.1	110	12	0.067	0.25	51.7	6.4	0.07	6.65	[81]
Ulu Sedili Besar	1.25	4.1	49	12	0.033	0.38	53.2	12.2	0.17	4.21	[81]
Indus River	1	10	160	12.4	0.030	0.38	168.3	15.6	0.17	1.10	[82]

^‡^ The values of *n* and *T* were not directly inferred from the corresponding references and thereby the values of these parameters were assumed as 0.020 s/m^1/3^ and 12.4 hours, respectively.

### Tidal range analysis

A sinusoidal tide was adopted in this study and tidal range (*TR*) was calculated as the difference in the average of water level between high (${\bar{\zeta }}_{high\;tide}$) and low (${\bar{\zeta }}_{low\;tide}$) tides:

$$TR={\bar{\zeta }}_{high\;tide}-{\bar{\zeta }}_{low\;tide}$$ (7)

**Fig 2:**
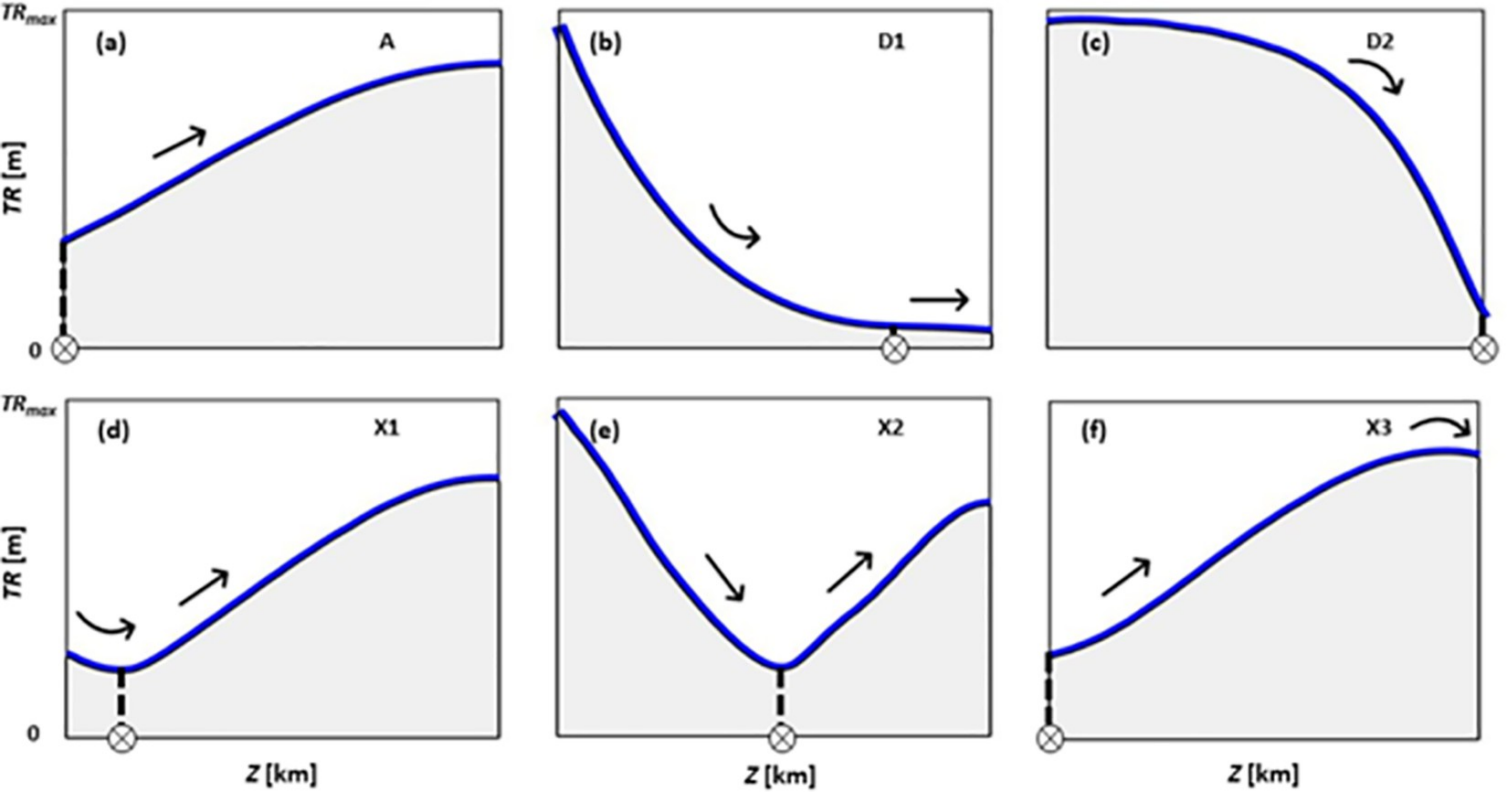
Conceptual (general) patterns of tidal range *TR* including (a) amplification (pattern A); (b) dampening with a concave-shaped decline (pattern D1); (c) dampening with a convex-shaped decline (pattern D2); (d) a mixed pattern (pattern X1) where *TR* decreases to the minimum before it rises to an upstream value higher than at the mouth; (e) a mixed pattern (pattern X2) where *TR* decreases up to the minimum and then starts rising with the upstream *TR* less than at the mouth; and (f) a mixed pattern (pattern X3) where *TR* increases to a maximum and then decreases. Circled crosses show the location of the minimum *TR* and arrows illustrate the direction of change in *TR*.

Tidal range was calculated along the centre nodes for all simulated cases. After analysing all tidal range patterns along the central transects, it was inferred that these patterns (blue lines in Fig 2) can take six general forms including amplification (A) (Fig 2A), dampening (D1, D2) (Fig 2B and 2C), and a mix of amplification and dampening (X1, X2, X3) (Fig 2D–2F). For all tidal range curves, the location of points with the minimum tidal range was depicted (circled crosses in Fig 2) to indicate potential trends in the tidal range upstream/downstream of these points (arrows in Fig 2). This provides important information regarding the influence of SLR on tidal dynamics. The tidal range patterns introduced are a breakdown analysis of hypersynchronous and hyposynchronous estuaries where, over the tidal limit, the tidal range constantly increases due to channel convergence or decreases due to the influence of bed and bank friction, respectively [34, 83, 84]. Generally, hypersynchronous and hyposynchronous conditions can characterise tide-dominated and wave-dominated systems, respectively [34, 83, 84].

Fig 2A shows an estuary where tidal range increases from the mouth towards the river head, potentially due to funnelling and/or tidal wave reflection. Thus, the location of the minimum tidal range for this case is at the mouth. Fig 2B and 2C shows the tidal range patterns in energy dissipative estuaries where tidal range decreases on a concave or convex direction, up to the point of the minimum tidal range, and after that, the rate of change in tidal range ((*TR*_*max*_−*TR*_*min*_)/*TR*_*max*_) is less than 5%, and the tidal range curve approaches equilibrium. Fig 2D and 2E illustrates cases where tidal range decreases up to the point of the minimum tidal range but then starts rising (e.g., due to resonance or reflection) towards the head with tidal range being higher (Fig 2D) or lower (Fig 2E) than at the mouth. Fig 2F demonstrates a case where tidal range rises from the mouth but then diminishes in the upstream potentially due to the presence of a strong river inflow.

## Results

**Fig 3:**
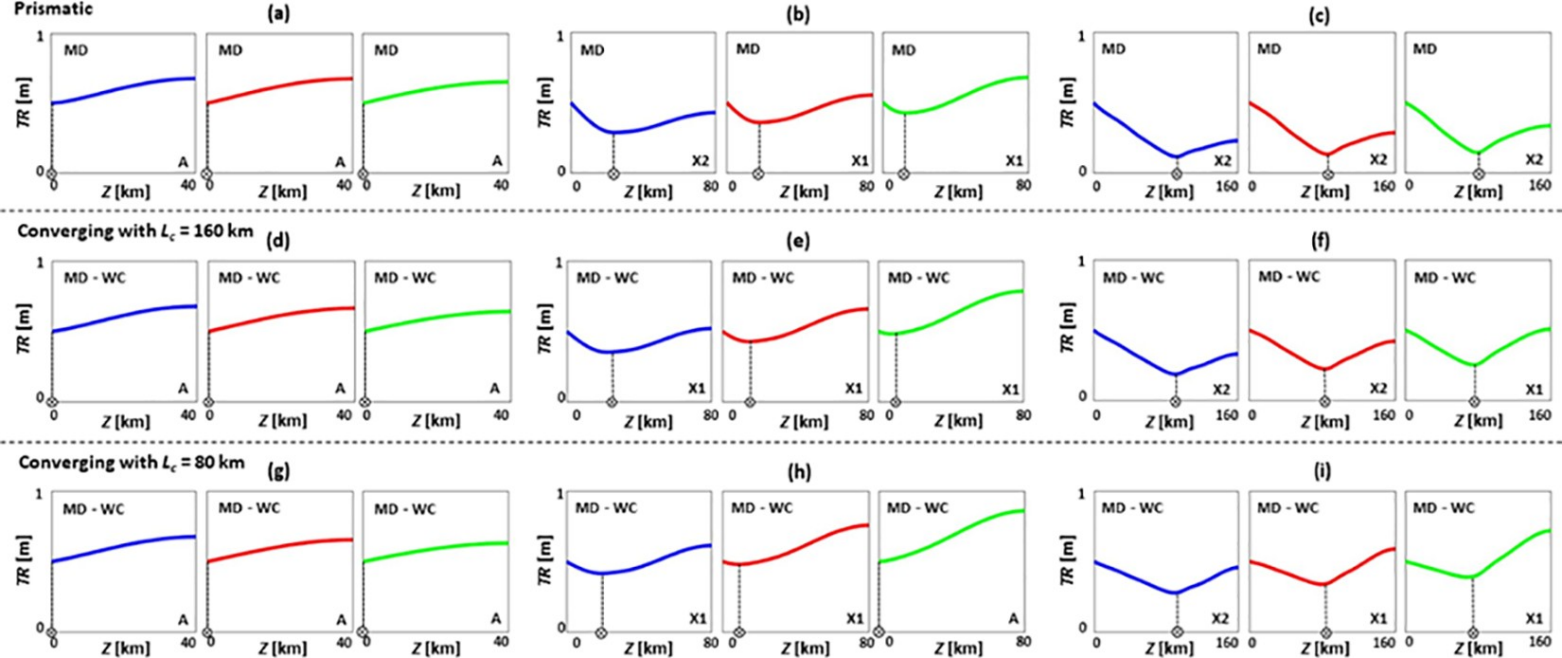
Tidal range *TR* patterns of prismatic (a-c), converging with *L_c_* = 160 km (d-f), and converging with *L_c_* = 80 km (g-i) estuaries under SLR, where*TR_0_* = 0.5 m, *Q*/*TP* = 0% (no river discharge), *n* = 0.03 s/m^*1/3*^, *h* = 5 m, and θ = 0°. (a), (d), and (g) are short estuaries (*Z* = 40 km); (b), (e), and (h) are moderate estuaries (*Z* = 80 km); and (c), (f), and (i) are long estuaries (*Z* = 160 km). Blue, red, and green colours show base case (no SLR), 1 m of SLR, and 2 m of SLR scenarios, respectively. Patterns A, X1, and X2 are the general *TR* patterns introduced in Fig 2. MD and WC refer to moderately dissipative and weakly converging estuaries, respectively.

**Fig 4:**
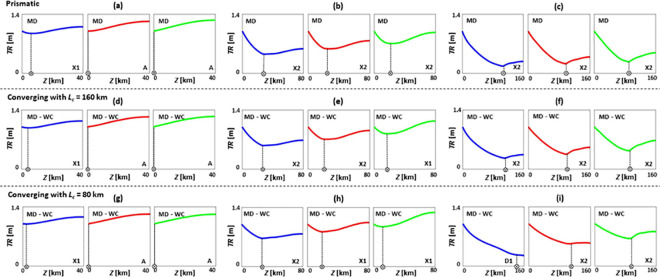
Tidal range *TR* patterns of prismatic (a-c), converging with *L*_*c*_ = 160 km (d-f), and converging with *L*_*c*_ = 80 km (g-i) estuaries under SLR, where *TR*_*0*_ = 1 m, *Q*/*TP* = 1% (low river discharge), *n* = 0.03 s/m^1/3^, *h* = 5 m, and θ = 0°. (a), (d), and (g) are short estuaries (*Z* = 40 km); (b), (e), and (h) are moderate estuaries (*Z* = 80 km); and (c), (f), and (i) are long estuaries (*Z* = 160 km). Blue, red, and green colours show base case (no SLR), 1 m of SLR, and 2 m of SLR scenarios, respectively. Patterns A, X1, X2, and D1 are the general *TR* patterns introduced in Fig 2. MD and WC refer to moderately dissipative and weakly converging estuaries, respectively.

**Fig 5:**
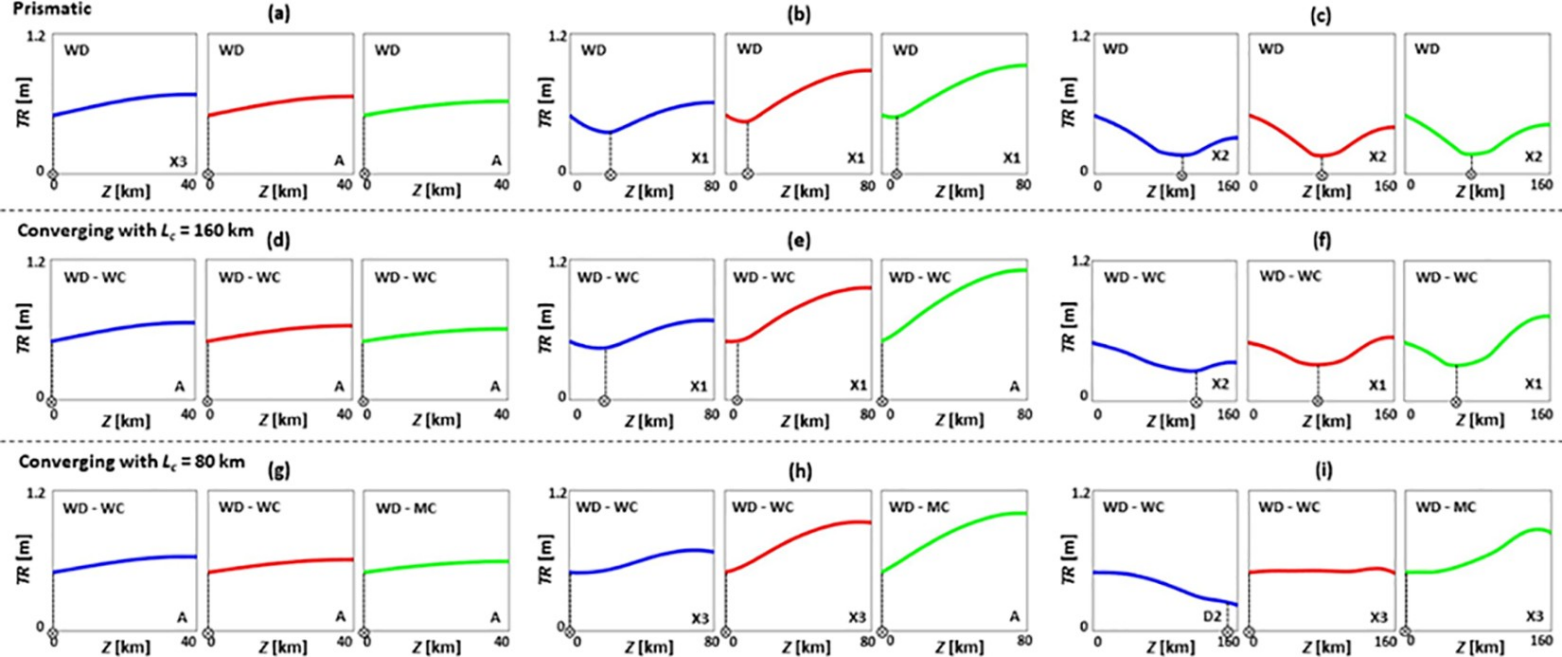
Tidal range *TR* patterns of prismatic (a-c), converging with *L*_*c*_ = 160 km (d-f), and converging with *L*_*c*_ = 80 km (g-i) estuaries under SLR, where *TR*_*0*_ = 0.5 m, *Q*/*TP* = 5% (medium river discharge), *n* = 0.015 s/m^1/3^, *h* = 5 m, and θ = 0°. (a), (d), and (g) are short estuaries (*Z* = 40 km); (b), (e), and (h) are moderate estuaries (*Z* = 80 km); and (c), (f), and (i) are long estuaries (*Z* = 160 km). Blue, red, and green colours show base case (no SLR), 1 m of SLR, and 2 m of SLR scenarios, respectively. Patterns A, X1, X2, X3, D1, and D2 are the general *TR* patterns introduced in Fig 2. WD, WC, and MC refer to weakly dissipative, weakly converging, and moderately converging estuaries, respectively.

**Fig 6:**
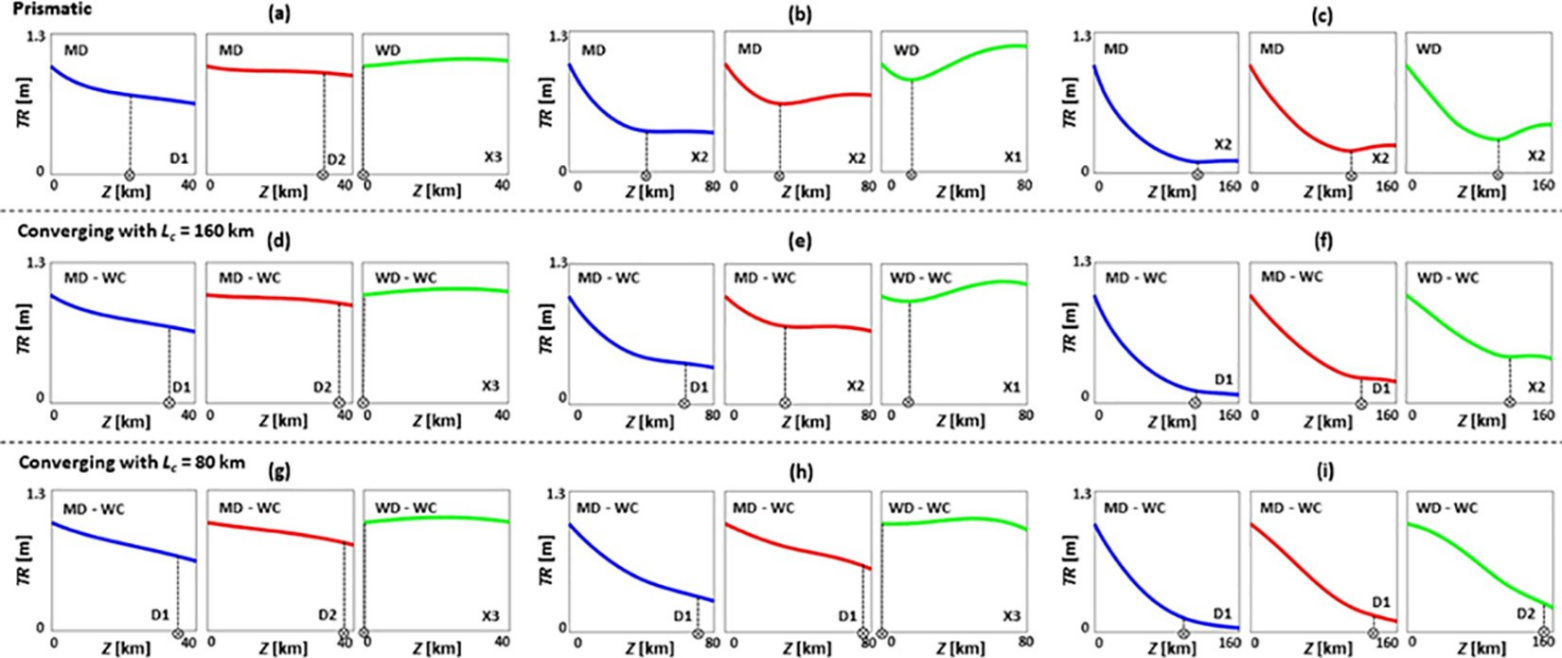
Tidal range *TR* patterns of prismatic (a-c), converging with *L*_*c*_ = 160 km (d-f), and converging with *L*_*c*_ = 80 km (g-i) estuaries under SLR, where *TR*_*0*_ = 1 m, *Q*/*TP* = 10% (high river discharge), *n* = 0.015 s/m^1/3^, *h* = 5 m, and θ = 0°. (a), (d), and (g) are short estuaries (*Z* = 40 km); (b), (e), and (h) are moderate estuaries (*Z* = 80 km); and (c), (f), and (i) are long estuaries (*Z* = 160 km). Blue, red, and green colours show base case (no SLR), 1 m of SLR, and 2 m of SLR scenarios, respectively. Patterns X1, X2, X3, D1, and D2 are the general *TR* patterns introduced in Fig 2. WD, MD, and WC refer to weakly dissipative, moderately dissipative, and weakly converging estuaries, respectively.

**Fig 7:**
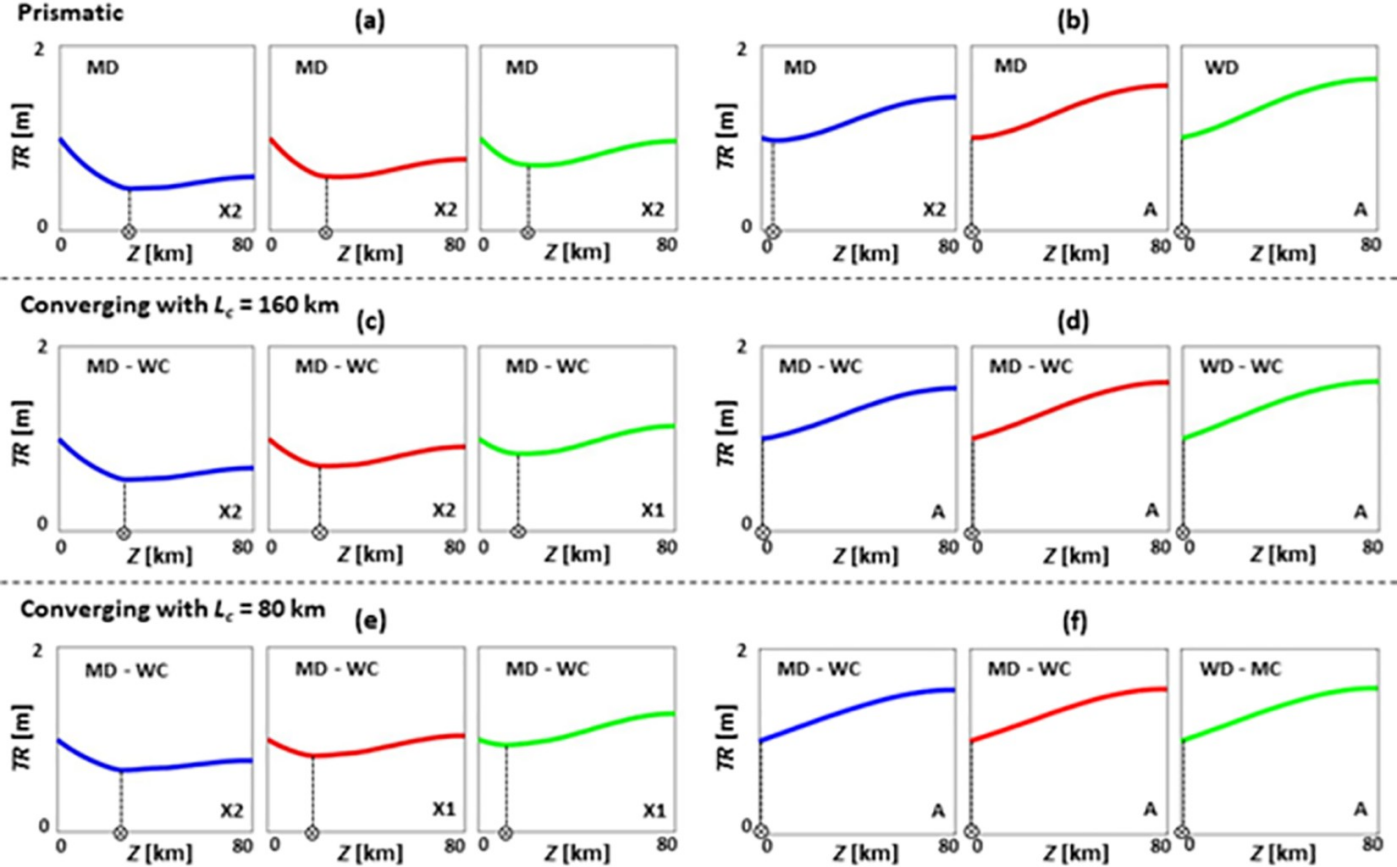
Influence of channel dredging on tidal range *TR* patterns of prismatic (a, b), converging with *L*_*c*_ = 160 km (c, d), and converging with *L*_*c*_ = 80 km (e, f) estuaries under SLR, where *TR*_*0*_ = 1 m, *Q*/*TP* = 1% (low river discharge), *Z* = 80 km, *n* = 0.03 s/m^1/3^, and θ = 0°. In (a), (c), and (e) *h* = 5 m, and in (b), (d), and (f) *h* = 10 m. Blue, red, and green colours show base case (no SLR), 1 m of SLR, and 2 m of SLR scenarios, respectively. Patterns X1, X2, and A are the general *TR* patterns introduced in Fig 2. WD, MD, WC, and MC refer to weakly dissipative, moderately dissipative, weakly converging, and moderately converging estuaries, respectively.

**Fig 8:**
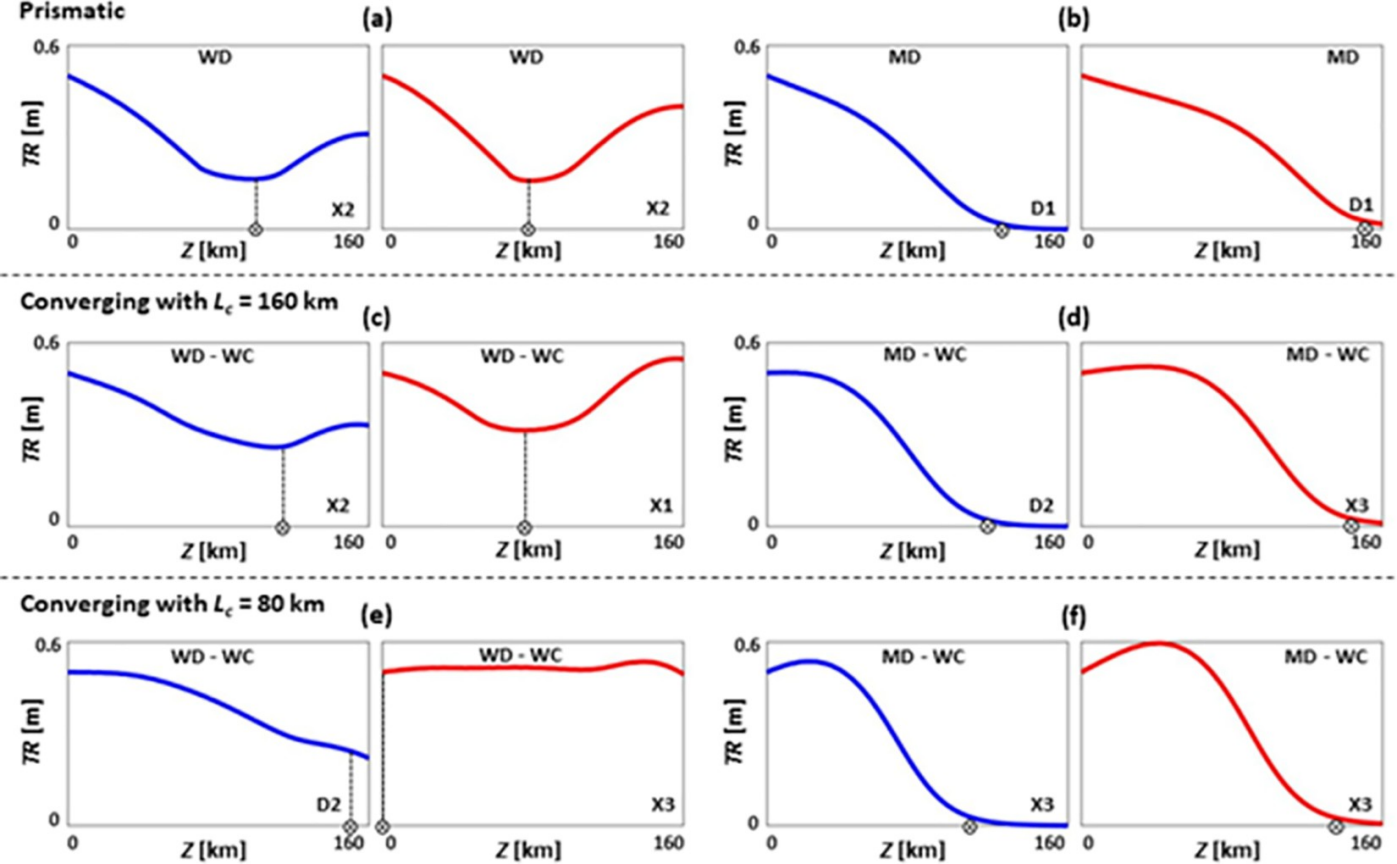
Influence of bed slope on tidal range *TR* patterns of prismatic (a, b), converging with *L*_*c*_ = 160 km (c, d), and converging with *L*_*c*_ = 80 km (e, f) estuaries under SLR, where *TR*_*0*_ = 0.5 m, *Q*/*TP* = 5% (medium river discharge), *Z* = 160 km, *n* = 0.015 s/m^1/3^, and *h* = 5 m. Cases in (a), (c), and (e) have flat beds (θ = 0°), and in (b), (d), and (f) have sloped beds (θ = 0.002°). Blue and red colours show base case (no SLR) and 1 m of SLR scenarios, respectively. Patterns X1, X2, X3, D1 and D2 are the general *TR* patterns introduced in Fig 2. WD, MD, and WC refer to weakly dissipative, moderately dissipative, and weakly converging estuaries, respectively.

To better understand how SLR may influence the tidal range dynamics of different estuary types, typical results of the numerical simulations are presented. These results provide insights into the influences that drive tidal range patterns and where these changes are likely to occur. The results presented in Figs 3–7 may be potentially reproduced using existing analytical and semi-analytical solutions for tidal amplitudes (e.g., [28, 38]), though they do not consider bed slope (Fig 8).

Here, results are presented for different estuary types, tidal ranges at the mouth, and river discharge conditions (e.g., no, low, medium, and high river discharge conditions). This analysis provides further understanding of the potential effects of river inflows and SLR on tidal range, which is an important indicator for tidal dynamics, sediment transport, water quality, and vegetation communities. The presented cases provide an overview of the typical estuarine tidal range responses to SLR, and the results of all cases are summarised in S1 Table. For all the provided result figures, blue, red, and green colours indicate the base case (no SLR), 1 m of SLR, and 2 m of SLR scenarios, respectively.

### Tidal range dynamics under sea level rise during no river discharge condition

Typical tidal range patterns of selected estuary types are presented without river discharge (*Q*/*TP* = 0%) and for different SLR scenarios (Fig 3). SLR increases tidal range for estuaries where *Z* = 80 or 160 km and shifts the spatial position of points with the minimum tidal range values. However, SLR has limited influence, or negligibly reduces, the tidal range of estuaries where *Z* = 40 km. These findings are consistent with [41], where it was indicated that the Potomac River estuary (*Z* ≃ 150 km), York River estuary (*Z* ≃ 85 km), and Rappahannock River estuary (*Z* ≃ 84 km), experience a tidal range amplification under 1 m of SLR, whereas the nearby Choptank River estuary (*Z* ≃ 42 km) undergoes a minimal tidal range attenuation under 1 m of SLR. Further, for all estuary lengths tested, the largest tidal change occurs at the 0–5% upstream length of the system where the reflective boundary is located.

Moreover, converging estuaries have higher tidal range values, in comparison to prismatic estuaries, as the energy is funnelled into a smaller cross-section and then reflected towards the mouth, leading to tidal range amplification, such as in the Severn River estuary [29] or the Hudson River estuary [85]. The increase in tidal range values is most evident in longer converging estuaries.

For longer estuaries (Fig 3C, 3F and 3I), tidal range is generally attenuated due to energy dissipation from frictional effects [7], however tidal amplification still occurs under SLR. Due to resonance, estuaries that are near one quarter of a wavelength (*L*/4) in length are highly vulnerable to tidal range amplification [39], and are sensitive to changes in estuary length or depth [19]. In these estuaries, resonance occurs since the tidal wave travel time from the mouth to the head and back to the mouth is nearly the same as the time between low and high tides. For estuaries where *Z* ≥ *L*/4 (i.e., *Z* = 80 and 160 km), SLR increases water depth, reduces frictional effects, and changes the wave celerity, leading to a shift in the spatial location of points with a minimum tidal range (circled crosses in Fig 3). For instance, as shown in Fig 3C, 3F and 3I, SLR moves the points of minimum tidal range towards the mouth (seaward), resulting in a rise in tidal amplitude at the upstream (landward) part of the estuary. The seaward displacement of the minimum tidal range in these idealised cases, is in line with the findings of [41] in the main stem of the Chesapeake Bay under 1 m of SLR. In a previous work [7], it was found that 1 m of SLR can increase the tidal range of estuaries close to resonance by 10–20%.

Further analysis on estuarine tidal range dynamics during zero river discharge conditions and for the whole range of tested parameters are presented in S1, S5, and S9 Tables. According to these tables, increasing tidal range at the mouth (*TR*_*0*_) would lead to a faster decay of tides. SLR can move the location of points with minimum tidal range upstream in the longest estuaries and for those with the largest roughness (*n* = 0.09 s/m^1/3^).

Tidal range can also increase or decrease with increasing estuary length, depending on *TR*_*0*_ and Manning’s *n*. When *TR*_*0*_ = 0.5 m, tidal range patterns for moderate and long prismatic estuaries would change under SLR (e.g., from X2 to X1). When *TR*_*0*_ = 1 m, tidal range patterns of prismatic estuaries do not alter under SLR, primarily remaining as X2 pattern. These patterns, however, would shift from X1 to A or X2 or X1 patterns under SLR when the estuaries are converging. When *TR*_*0*_ = 4 m, short prismatic estuaries would experience a change in their tidal range patterns either from X1 to A or from X2 to X1, depending on the Manning’s *n*.

### Tidal range dynamics under sea level rise during low river discharge condition

In this section, typical tidal range responses of selected estuary types to SLR are presented in Fig 4, under low river discharge conditions (*Q*/*TP* = 1%). While short estuaries (Fig 4A, 4D and 4G) are dominated by tidal range amplification over the majority of the length due to reflection at the head, they experience minor tidal dampening in the downstream sections for cases without SLR. However, SLR can shift tidal range patterns of these cases from X1 to A. For estuaries close to resonance length (Fig 4B, 4E and 4H), converging estuaries with *L*_*c*_ = 80 km retain the highest tidal range values and experience rapid changes in tidal range patterns under SLR. SLR moves the points of minimum tidal range of these estuaries closer to the mouth and resonance state, bringing about a rise in tidal range, with the upstream range higher than the mouth (X1 pattern). However, tidal range patterns of prismatic estuaries do not change under 1 m and 2 m of SLR, remaining as X2 patterns for all scenarios. The tidal range pattern of converging estuaries with *L*_*c*_ = 160 km only alters under SLR of 2 m, shifting from X2 to X1. Tidal range patterns of long estuaries (Fig 4C, 4F and 4I) are mainly dominated by X2 patterns and SLR moves the points of minimum tidal range seaward. Therefore, a weak energy reflection still exists in the upstream end of these long estuaries, strengthening tidal range amplification, as in the lower bay of the Delaware estuary [42]. Additionally, the maximum tidal change appears at the 0–25% upstream length of the short estuaries, while it occurs at the 0–5% upstream length of the moderate and long systems.

Further, from comparing cases with *Q*/*TP* = 0% and 1%, it is evident that adding river discharge leads to a decline in tidal range for most cases. This decrease in tidal range under river discharge condition is in the range of 0–78%, 0–93%, and 0–98% for prismatic, converging with *L*_*c*_ = 160 km, and converging with *L*_*c*_ = 80 km estuaries, respectively. This is also valid in real-world estuaries, such as the Scheldt estuary, where river discharge is responsible for tidal dampening in the upstream part of the estuary [86]. The location of maximum tidal change under an incremental change in river flow was found to be the upstream area, such as in the Modaomen estuary [87, 88]. Thus, it is important to consider the influence of river inflow variations on estuarine tidal range dynamics and SLR studies.

Further findings on estuarine tidal range responses to SLR during low river discharge conditions for more simulated cases are presented in S2, S6, and S10 Tables. As per these tables, cases with higher roughness are dominated by X2 and D1 patterns. Regardless of the estuary type, pattern A mainly occurs in short estuaries with lower *TR*_*0*_ and *n* values. In longer estuaries with higher *TR*_*0*_ and *n* values, SLR can move the points of the minimum tidal range landward, but the converse is valid for other cases (i.e., long estuaries with low *TR*_*0*_ and *n* values).

In prismatic estuaries, SLR alters the tidal range patterns of moderate (for all friction) and long (with low and high friction) estuaries when *TR*_*0*_ = 0.5 m; short (with moderate and high friction), moderate (with high friction), and long (with high friction) estuaries when *TR*_*0*_ = 1 m; and short (with low and moderate friction) and moderate (with high friction) estuaries when *TR*_*0*_ = 4 m. In converging estuaries with *L*_*c*_ = 160 km, SLR changes the tidal range patterns of short (with high friction) and moderate (for all friction) estuaries when *TR*_*0*_ = 0.5 m; short (with moderate and high friction), moderate (for all friction), and long (with high friction) estuaries when *TR*_*0*_ = 1 m; and short (with moderate friction) and moderate (with low friction) estuaries when *TR*_*0*_ = 4 m. In converging estuaries with *L*_*c*_ = 80 km, SLR shifts the tidal range patterns of short (with high friction), moderate (with moderate and high friction), and long (with moderate friction) estuaries when *TR*_*0*_ = 0.5 m; short (with moderate and high friction), moderate (with low and moderate friction), and long (with moderate friction) estuaries when *TR*_*0*_ = 1 m; and short (with low and moderate friction) and long (with moderate friction) estuaries when *TR*_*0*_ = 4 m.

### Tidal range dynamics under sea level rise during medium river discharge condition

The tidal range responses of different estuary types to SLR are presented in Fig 5, for medium river discharge conditions (*Q*/*TP* = 5%). Without SLR, only short converging estuaries (Fig 5D and 5G) with *n* = 0.015 s/m^1/3^ experience a tidal range amplification due to energy convergence and reflection at the estuary head. For prismatic estuaries with *Z* = 80 km (Fig 5B), the tidal range pattern is independent of SLR and always remains as pattern X1. For a converging estuary with *L*_*c*_ = 160 km and *Z* = 80 km (Fig 5E), only a SLR of 2 m can change the tidal range pattern from X1 for the base case and 1 m SLR, to pattern A under SLR of 2 m. Converging estuaries with *L*_*c*_ = 80 km and *Z* = 80 km (Fig 5H) undergo tidal range amplification potentially due to the presence of a reflective boundary, with the largest tidal range occurring at the head [19]. Longer estuaries (Fig 5C, 5F and 5I) do not experience progressive tidal range amplification (pattern A) under SLR. In long prismatic estuaries (Fig 5C), the tidal range pattern is unaffected by SLR and is consistently X2. In long converging estuaries with *L*_*c*_ = 160 km (Fig 5F), tidal range patterns shift from X2 to X1 under SLR, as SLR moves the points of minimum tidal range seaward, leading to a tidal range increase in the landward direction. The tidal range pattern of long converging estuaries with *L*_*c*_ = 80 km (Fig 5I) changes significantly from D2 to X3 under SLR. In this case, SLR shifts the system from hyposynchronous to hypersynchronous condition, and a SLR of 2 m could even change the system from weakly converging to moderately converging with further tidal range amplification. Further, the maximum tidal range change appears at the 0–15% upstream length of the short estuaries, while it occurs at the 0–5% upstream length of the moderate and long systems.

Further analysis regarding the tidal range response of different estuary types to SLR during medium river discharge conditions are presented in S3, S7, and S11 Tables. According to these tables, pattern A (increasing tidal range amplification) rarely occurs due to the presence of a strong river discharge. The applied river inflow at the head can act as an additional source of frictional effects [38], reducing the tidal range in the upstream reaches [86]. In longer estuaries with higher *TR*_*0*_ and *n* values, SLR can move the points of minimum tidal range landward but would move them seaward for other cases (i.e., long estuaries with low *TR*_*0*_ and *n* values). Further, more cases (often with higher *TR*_*0*_ and *n* values) demonstrate X2 and D1 patterns in comparison to similar cases during zero or low river discharge conditions due to increasing river inflows that attenuate the propagating tidal waves [30, 31]. This is in good agreement with real-world estuaries, such as the Fraser River estuary, Saint Lawrence River estuary, and Saint John River estuary where increasing river inflow would reduce upstream tidal range in comparison to downstream values [89].

In prismatic estuaries, SLR changes the tidal range patterns of short (with low and moderate friction), moderate (with moderate friction) and long (with moderate friction) estuaries when *TR*_*0*_ = 0.5 m; short (with moderate friction), moderate (with low and moderate friction), and long (with moderate friction) estuaries when *TR*_*0*_ = 1 m; and short (with low friction) and moderate (with moderate friction) estuaries when *TR*_*0*_ = 4 m. In converging estuaries with *L*_*c*_ = 160 km, SLR varies the tidal range patterns of short (with moderate friction), moderate (with low and moderate friction), and long (with low and moderate friction) estuaries when *TR*_*0*_ = 0.5 m; short (with moderate friction), moderate (with low and moderate friction), and long (with low friction) estuaries when *TR*_*0*_ = 1 m; and long (with low friction) estuaries when *TR*_*0*_ = 4 m. In converging estuaries with *L*_*c*_ = 80 km, SLR alters the tidal range patterns of short (with moderate friction), moderate (with low and moderate friction), and long (with low and moderate friction) estuaries when *TR*_*0*_ = 0.5 m; moderate (with low friction) and long (with low friction) estuaries when *TR*_*0*_ = 1 m; and moderate (with low friction) estuaries when *TR*_*0*_ = 4 m.

### Tidal range dynamics under sea level rise during high river discharge condition

Typical tidal range patterns of selected estuaries due to SLR are illustrated in Fig 6, for high river discharge conditions (*Q*/*TP* = 10%). In short estuaries (Fig 5A, 5D and 5G), tidal range is dampened in a concave direction (D1 pattern) for base cases but in a convex direction (D2 pattern) under 1 m of SLR. However, a SLR of 2 m amplifies the tidal range over most of the length of short estuaries due to tidal wave reflection at the head. In longer estuaries (*Z* = 80 and 160 km), strong friction induced by river discharge can induce dampening of the tides and eliminate reflection or resonance at the head [10] for most base cases and 1 m SLR cases. Under 2 m of SLR, a weak tidal wave reflection appears in the upper reaches. This outcome is consistent with the findings of [28] who indicated that reflection becomes important in 1/3 of the most upstream part of closed end estuaries. For instance, Fig 6H shows a case where tidal range can shift from fully attenuated for a base case or a case with 1 m of SLR to amplifying and then dampening for a case with 2 m of SLR. This increase in tidal range can produce higher water levels as river discharges cannot drain as rapidly as before, increasing the risk of inundation especially in the upstream part of the estuary [89]. In Fig 6H, the minimum and maximum water depths are 5.5 and 8.1 m, 6.5 and 8.2 m, and 7.5 and 8.6 m for the base case, with 1 m of SLR, and with 2 m of SLR, respectively. Additionally, the maximum tidal change appears at the most 0–45% upstream length of the short estuaries, while it occurs at the most 0–5% upstream length of the moderate and long systems.

Further analysis regarding the tidal range responses of different estuary types to SLR during high river discharge conditions are summarised in S4, S8, and S12 Tables. As per these tables, tidal range patterns of most cases are dominated by D1 and X2 patterns due to the dampening effect under significant upstream river discharge. In most estuaries, SLR moves the points of minimum tidal range upstream, contrasting with tidal range responses during zero or low river discharge conditions. The only exceptions are short and moderate estuaries with very low friction (*n* = 0.015 s/m^1/3^) for which SLR shifts these points downstream.

## Discussion

As shown for the three estuary types, SLR can increase the water depth, decrease friction, and change the distribution of points with minimum tidal range in an estuary. For most estuaries, this will lead to an amplified tidal range, altering the spatial structure of tidal currents [10]. Any variations in tidal range and currents can then bring about changes to flood risk [50], extractable tidal energy [90], mixing and circulation patterns [91], sediment dynamics [23], water quality [92], and vegetation communities [26].

Among estuary types tested, tidal range amplification is more evident in converging estuaries where tides can be amplified due to energy convergence. It has been analytically indicated that tidal range can be increased if *L*_*c*_≪3*πhω*/8*C*_*D*_*U*К, where *ω* is tidal frequency, *C*_*D*_ is the drag coefficient (*C*_*D*_ = *gn*^2^*h*^−1/3^), *U* is tidal current velocity amplitude, and К is the wave number [40, 53, 85]. Therefore, a moderately/strongly converging estuary with smaller values of *L*_*c*_ has a higher chance of tidal range amplification. Further, the strength of the upstream river discharge is important as it acts as an additional source of friction, reducing upstream tidal range. In a real estuary with geomorphic adaptations, river discharge can also change the overall estuary shape and thereby, tidal dynamics. For instance, the Yangtze River estuary has a prismatic shape during medium river discharge conditions, but shifts to a more convergent shape during low river discharge conditions [93]. Moreover, for the estuaries tested, the location of maximum changes in tidal range due to an incremental change in river inflow was found to be in the upstream area, which is in line with the Scheldt [86], Modaomen [87, 88], Yangtze [87], Fraser River [51], and Columbia River [51] estuaries under different river inflow conditions.

Tidal range amplification may also occur in estuaries with extensive engineered riverbanks, including levees, dykes, weirs, and vertical retaining walls. Propagating tidal waves may be reflected seaward by these structures, further increasing tidal range, such as in the Ems [94] and Scheldt [28] estuaries. These structures may disconnect intertidal areas which would elsewise add natural friction and energy storage, thereby increasing inundation risks [8]. Further, partial reflection may also occur mid-estuary due to abrupt changes in channel width/depth (e.g., under SLR), creating a maximum tidal range [95, 96]. The tidal range may reduce after the point of partial reflection, such as in the Columbia River estuary [95, 96].

Dredging is also common in many estuaries worldwide to maintain or increase navigation [97]. Although SLR and channel dredging/deepening are different processes, they both may lead to tidal range amplification (for details, see [98]). To conceptually illustrate the likely effect of channel dredging on estuarine tidal range, Fig 7 depicts tidal range patterns of moderate estuaries (*Z* = 80 km) with a minor river discharge (*Q*/*TP* = 1%) when *TR*_*0*_ = 1 m, *n* = 0.03 s/m^1/3^, SLR = 0, 1, and 2 m, and *h* = 5 and 10 m (assuming the entire estuary is dredged). As is clear from Fig 7, none of the cases where *h* = 5 m (Fig 7A, 7C and 7E) experience an increasing tidal range amplification even under 1 m or 2 m of SLR, though reflection still exists in the upstream reaches due to the presence of protective structures. For the cases mimicking channel deepening, where *h* = 10 m (Fig 7B, 7D and 7F), tidal range amplification (pattern A) occurs in almost all estuaries due to the reduced friction under increasing mean water levels and the seaward displacement of points with a minimum tidal range. These cases could be representatives of channels that are dredged for navigation and may subsequently experience tidal range amplification, as in the Delaware Bay [99] or the Hudson River [85].

In general, there are more changes in tidal range patterns when the depth of an estuary increases from 5 m to 6 m or 7 m compared to depth changes from 10 m to 11 m or 12 m under SLR. In estuaries with depths of 10–12 m, the tidal range patterns are often A, X1, or X2, except where *Q*/*TP* = 5–10% and *n* = 0.09 s/m^1/3^ with D1 and D2 patterns.

Although a zero-bed slope is widely accepted as an assumption in estuarine analytical studies [39], the estuary depth may decrease exponentially from the mouth to the upstream tidal limit, particularly for systems with strong topographic relief [39]. The influence of sloped beds on tides is briefly discussed below, as they cannot be replicated analytically.

While it is believed that the limit of tidal intrusion is often related to dams and weirs, a sloped bed is potentially sufficient to fully dampen the tides without any additional physical obstacles [100]. To show the influence of bed slope, Fig 8 illustrates how tidal ranges of long estuaries (*Z* = 160 km) with a strong river discharge (*Q*/*TP* = 5%) may alter under SLR when *TR*_*0*_ = 0.5 m, *n* = 0.015 s/m^1/3^, *h* = 5 m, and θ = 0° and 0.002°. In estuaries with θ = 0° (Fig 8A, 8C and 8E), tidal range is often maintained higher than in cases with θ = 0.002° (Fig 8B, 8D and 8F), as the latter presents higher friction due to a reduced water depth. In estuaries with sloped beds, the shallower depth may eliminate the tidal range amplification occurring in the upstream part of the estuary [100]. For a prismatic estuary, tidal range pattern changes from a mix of amplification and dampening for a flat bed to purely dampening for a sloped bed (Fig 8A and 8B). For converging estuaries (Fig 8C–8F), tidal range pattern generally remains a mix of dampening and amplification, but tidal range tends to zero in the upstream parts for cases with θ = 0.002° (Fig 8D and 8F). Under SLR, points of minimum tidal range move upstream. As such, bed slope is an important component in estimating estuarine tidal dynamic responses to SLR, although it has largely been disregarded in most analytical research. If any estuary experiences a change in its geometry (e.g., depth, slope, shape) under SLR (e.g., due to an altered sediment transport dynamics), its tidal range response to SLR may vary significantly.

It is worth noting that the results of the idealised models presented in this study are based on several assumptions and further modifications should be considered. For instance, the immediate changes in estuarine geomorphology under SLR have been disregarded although mobile sediments are present in most estuaries. Further, the models did not include the adjacent low-lying intertidal areas that could be periodically inundated now and under SLR. For instance, if these areas are inundated over a tide cycle, they can add natural water storage volume and friction, thereby attenuating the tides (for details, see [42]).

Generally, estuaries can be classified into two groups: (a) with entrance restrictions (e.g., due to formation of a bar or spit across the entrance [101]) or (b) no entrance restrictions [102]. Unrestricted entrance estuaries can have either a single wide-open entrance or a deltaic coastal plain with a few distributaries [102]. The present study only considered unrestricted entrance estuaries with a single wide mouth. Further, real-world estuaries may not be fully convergent to the upstream tidal limit but approach a constant width condition (e.g., [100]) at some distance from the tidal limit. In this case, the tidal range response in the upstream portions of the estuary may behave more like a prismatic estuary. Not considering a continental shelf domain may also become important where tides interact with the surrounding bathymetry and generate overtides [103] which could become significant relative to the main constituents [31].

As only M2 tides were considered, the modelling results provide a reasonable proxy for tidal range [104, 105]. For instance, doubling M2 is a reasonable approximation of tidal range but this ratio may change due to the presence of other tidal constituents and for different geographical locations [106]. Considering only M2 tide may also eliminate the nonlinear interactions between different tidal constituents, arising from the fact that the friction term in the momentum equation is based on a quadratic law that results in nonlinear interactions, generating tidal asymmetry [107, 108]. The frictional interactions between tidal constituents and how their ratios may shift in the landward direction have not been considered in this study, though they may affect the tidal range [109]. Further, as reflection is a function of frequency, diurnal tidal constituents may have different reflection characteristics, influencing the tidal range [110].

Other energy drivers, including wind and waves, were not considered in this study for simplicity. To allow a generalised and latitude-independent study, the Coriolis force was disregarded. Since the geomorphology of estuaries responds to SLR slower compared to the instantaneous response of tidal range dynamics (up to 2 orders of magnitude [21]), geomorphic adaptations were not considered here. The influence of salinity intrusion/stratification is not discussed within this study and all estuaries were assumed to be well-mixed. However, salinity stratification can be expected in estuaries with large river inflows, affecting the tidal dynamics.

## Conclusions

Accelerating SLR will have significant environmental and socio-economic impacts on hundreds of millions of inhabitants living within estuarine catchments. As estuarine tidal range responses to SLR are complex and site specific, managers and policy makers require accurate information to develop sustainable management plans for estuaries. The primary motivation of this study is to expand upon existing analytical and semi-analytical studies and provide a parameter space study to investigate the combined influence of river inflows and SLR on tides. To this end, over 1800 idealised estuarine hydrodynamic cases were simulated to provide further understanding of the potential impacts of SLR and river inflows on tidal range dynamics of various estuary types with different boundary conditions (i.e., weakly, moderately, and strongly dissipative as well as prismatic, weakly, and moderately converging estuaries).

The modelling results indicate that SLR can amplify the tidal range in different estuaries and shift the location of the minimum tidal range values–except in short estuaries with low tidal range at the mouth where SLR generally reduces the tidal range. SLR may also change estuarine tidal range patterns from fully dampening to a mix of amplification and dampening, or from a mixed pattern to a progressively amplified pattern.

The estuary types that are likely to experience significant tidal range amplification under SLR are summarised in Table 3. The outcomes of this study are useful to predict SLR impacts in estuaries, particularly where advanced hydrodynamic modelling is not currently available. However, additional site specific investigations are required to understand the implications of these findings at individual sites.

**Table 3:** Various estuary types with different boundary conditions that are likely to experience increasingly tidal range amplification under SLR.

*Q*/*TP* (%)	Estuary type	Estuary length (km)	*TR*_*0*_ (m)	Manning’s *n* (s/m^1/3^)
0	Prismatic	40	1	0.03
			4	0.015, 0.03
		80	0.5, 4	0.015
		160	0.5	0.015, 0.09
	Converging with *L*_*c*_ = 160 km	40	0.5	0.09
			1, 4	0.03
		80	0.5, 4	0.015
			1	0.015, 0.03
		160	0.5	0.03
			1	0.015
	Converging with *L*_*c*_ = 80 km	40	0.5	0.09
			1, 4	0.015, 0.03
		80	0.5, 1	0.015, 0.03
		160	0.5	0.03
			1	0.015
1	Prismatic	40	1	0.03, 0.09
			4	0.015, 0.03
		80	0.5	0.015, 0.03, 0.09
			1, 4	0.09
		160	0.5	0.015, 0.09
			1	0.09
	Converging with *L*_*c*_ = 160 km	40	0.5	0.09
			1	0.03, 0.09
			4	0.03
		80	0.5, 1	0.015, 0.03, 0.09
			4	0.015
		160	1	0.015
	Converging with *L*_*c*_ = 80 km	40	0.5	0.09
			1	0.03, 0.09
			4	0.015, 0.03
		80	0.5	0.03, 0.09
			1	0.015, 0.03
		160	0.5, 1, 4	0.03
5	Prismatic	40	0.5, 1, 4	0.015, 0.03
		80	0.5, 1, 4	0.015, 0.03
		160	0.5, 1	0.03
	Converging with *L*_*c*_ = 160 km	40	0.5, 1	0.03
		80	0.5, 1	0.015, 0.03
		160	0.5	0.015, 0.03
			1, 4	0.015
	Converging with *L*_*c*_ = 80 km	40	0.5	0.03
		80	0.5	0.015, 0.03
			1, 4	0.015
		160	0.5	0.015, 0.03
			1	0.015
10	Prismatic	40	1	0.015
		80	0.5, 1, 4	0.015
		160	4	0.015
	Converging with *L*_*c*_ = 160 km	40	1	0.015
		80	0.5, 1	0.015
		160	0.5, 1	0.015
	Converging with *L*_*c*_ = 80 km	40	1	0.015
		80	0.5, 1	0.015
		160	1	0.015

## Supporting information

S1 Table A summary of estuarine tidal range responses to SLR during no river discharge conditions (*Q*/*TP* = 0%) for prismatic estuaries. (PDF)

S2 Table A summary of estuarine tidal range responses to SLR during low river discharge conditions (Q/TP = 1%) for prismatic estuaries. (PDF)

S3 Table A summary of estuarine tidal range responses to SLR during medium river discharge conditions (Q/TP = 5%) for prismatic estuaries. (PDF)

S4 Table A summary of estuarine tidal range responses to SLR during high river discharge conditions (Q/TP = 10%) for prismatic estuaries. (PDF)

S5 Table A summary of estuarine tidal range responses to SLR during no river discharge conditions (Q/TP = 0%) for converging estuaries with *L*_*c*_ = 160 km. (PDF)

S6 Table A summary of estuarine tidal range responses to SLR during low river discharge conditions (Q/TP = 1%) for converging estuaries with *L*_*c*_ = 160 km. (PDF)

S7 Table A summary of estuarine tidal range responses to SLR during medium river discharge conditions (Q/TP = 5%) for converging estuaries with *L*_*c*_ = 160 km. (PDF)

S8 Table A summary of estuarine tidal range responses to SLR during high river discharge conditions (Q/TP = 10%) for converging estuaries with *L*_*c*_ = 160 km. (PDF)

S9 Table A summary of estuarine tidal range responses to SLR during no river discharge conditions (Q/TP = 0%) for converging estuaries with *L*_*c*_ = 80 km. (PDF)

S10 Table A summary of estuarine tidal range responses to SLR during low river discharge conditions (Q/TP = 1%) for converging estuaries with *L*_*c*_ = 80 km. (PDF)

S11 Table A summary of estuarine tidal range responses to SLR during medium river discharge conditions (Q/TP = 5%) for converging estuaries with *L*_*c*_ = 80 km. (PDF)

S12 Table A summary of estuarine tidal range responses to SLR during high river discharge conditions (Q/TP = 10%) for converging estuaries with *L*_*c*_ = 80 km. (PDF)
