# Supplementary material for: Estuarine tidal range dynamics under rising sea levels
Source: PLoS One. 2021 Sep 20;16(9):e0257538. doi: 10.1371/journal.pone.0257538 (PMC8452028; doi:10.1371/journal.pone.0257538)
Supplement: S1 Table — (PDF) [file pone.0257538.s001.pdf]

Supporting information for:

Estuarine tidal range dynamics under rising sea levels

Danial Khojasteh [1] , Shengyang Chen [1], Stefan Felder [1],

Valentin Heimhuber [1], William Glamore [1]

[1] *Water Research Laboratory, School of Civil and Environmental Engineering, UNSW Sydney, NSW 2093, Australia*

Corresponding author: Danial Khojasteh

Email addresses: danial.khojasteh@unsw.edu.au; s.chen@wrl.unsw.edu.au; s.felder@unsw.edu.au; v.heimhuber@unsw.edu.au; w.glamore@wrl.unsw.edu.au

In this study, 1836 hydrodynamic simulations were performed to present a relevant parameter space analysis and gain a better understanding on how tidal range dynamics of different estuary types (i.e., prismatic, weakly converging, and moderately converging, as illustrated in Fig. 1) may respond to future sea level rise (SLR). To this end, several parameters have been varied across the simulation cases including tidal range at the mouth (TR_0), estuary length (Z), estuary depth (h), Manning's roughness (n), bed slope (θ), river inflow over tidal prism ratio (Q/TP), and SLR scenarios. For all cases, the water surface elevations in X and Y directions (see coordinate system in Fig. 1), were extracted at every time step (each 15 minutes) and at each central node along the estuary for a total running period of 30 days. The tidal range (the difference between high and low tide levels) was then evaluated, and it was found that there are six general tidal range patterns for all cases including amplification (A), dampening (D1, D2), and a mix of amplification and dampening (X1, X2, X3) (see Fig. 2). The locations of points with minimum tidal range values were identified as they can provide information on how tidal range patterns may shift under SLR. Below tables present the changes in locations of points with minimum tidal range and the patterns of tidal range in response to SLR for different estuary types examined, as per no (Q/TP = 0%), low (Q/TP = 1%), medium (Q/TP = 5%), and high (Q/TP = 10%) river discharge conditions.

S1 to S4 Tables are for prismatic estuaries, S5 to S8 Tables are for converging estuaries with L_c = 160 km, and S9 to S12 Tables are for converging estuaries with L_c = 80 km. In these tables, three different estuary lengths (Z = 40, 80, and 160 km) are introduced as short, moderate, and long estuaries, respectively. Further, Manning's roughness of n = 0.015, 0.03, and 0.09 s/m$^{1/3}$ are represented as low, mod (moderate), and high friction, respectively. Various tested tidal ranges at the mouth TR_0 = 0.5, 1, and 4 m are presented as low, medium, and high tidal

ranges, respectively. All below cases have flat beds (θ = 0°), and initial water depth of h = 5 m. In each row, same highlighting colours show similar patterns.

As an example, in S1 Table, for a long estuary (Z = 160 km) with low friction (n = 0.015 s/m$^{1/3}$) and low entrance tidal range (TR_0 = 0.5 m), location of point with the minimum tidal range along the estuary is initially located at 81.65 km away from the mouth but will move downstream by 10% (8.16 km) and 13% (10.61 km) under 1 m and 2 m of SLR, respectively. The pattern of tidal range is initially X2 which shows that the tidal range decreases up to the minimum tidal range point and then starts rising, with the upstream range less than that of the mouth (see Fig. 2(e)). This pattern will not change under 1 m of SLR but will shift to X1 pattern under 2 m of SLR, indicating that the tidal range will decrease up to the minimum tidal range point and then starts rising, with the upstream range higher than that of the mouth (see Fig. 2(d)).

S1 Table. A summary of estuarine tidal range responses to SLR during no river discharge conditions (Q/TP = 0%) for prismatic estuaries.

		Short estuary (Z = 40 km)			Moderate estuary (Z = 80 km)			Long estuary (Z = 160 km)		
**Initial tidal range**	**Tidal range response**	**Low friction (n = 0.015 s/m$^{1/3}$)**	**Mod friction (n = 0.03 s/m$^{1/3}$)**	**High friction (n = 0.09 s/m$^{1/3}$)**	**Low friction (n = 0.015 s/m$^{1/3}$)**	**Mod friction (n = 0.03 s/m$^{1/3}$)**	**High friction (n = 0.09 s/m$^{1/3}$)**	**Low friction (n = 0.015 s/m$^{1/3}$)**	**Mod friction (n = 0.03 s/m$^{1/3}$)**	**High friction (n = 0.09 s/m$^{1/3}$)**
Low (TR_0 = 0.5 m)	Location of minimum tidal range	Entrance	Entrance	14.81 km away from the entrance for base case – it moves downstream by 11% and 41% for 1 and 2 m SLR, respectively	13.55 km away from the entrance for base case – it moves downstream by 55% and 100% for 1 and 2 m SLR, respectively	22.92 km away from the entrance for base case – it moves downstream by 31% and 56% for 1 and 2 m SLR, respectively	41.86 km away from the entrance for base case – it moves downstream by 8% and 14% for 1 and 2 m SLR, respectively	81.65 km away from the entrance for base case – it moves downstream by 10% and 13% for 1 and 2 m SLR, respectively	85.86 km away from the entrance for base case – it moves downstream by 5% and 12% for 1 and 2 m SLR, respectively	40.38 km away from the entrance for base case – it moves upstream by 124% and 157% for 1 and 2 m SLR, respectively
	Tidal range pattern	A	A	X2	X1 but SLR of 2m takes cases to A	X2 but SLR takes cases to X1	X2	X2 but SLR of 2m takes cases to X1	X2	D1 but SLR takes cases to X2
Medium (TR_0 = 1 m)	Location of minimum tidal range	Entrance	5.54 km away from the entrance for base case – it moves downstream at the entrance	21.26 km away from the entrance for base case – it moves downstream by 17% and 24% for 1 and 2 m SLR, respectively	19.97 km away from the entrance for base case – it moves downstream by 42% and 54% for 1 and 2 m SLR, respectively	33.42 km away from the entrance for base case – it moves downstream by 12% and 26% for 1 and 2 m SLR, respectively	47.28 km away from the entrance for base case – it moves downstream by 1% and 6% for 1 and 2 m SLR, respectively	87.46 km away from the entrance for base case – it moves downstream by 8% and 18% for 1 and 2 m SLR, respectively	90.47 km away from the entrance for base case – it moves downstream by 3% and 11% for 1 and 2 m SLR, respectively	97.50 km away from the entrance for base case – it moves upstream by 1% and 3% for 1 and 2 m SLR, respectively
	Tidal range pattern	A	X1 but SLR takes cases to A	X2	X1	X2	X2	X2	X2	X2
High	Location of	6.75 km away from the	14.00 km away from the	27.30 km away from the	33.83 km away from the	45.07 km away from the	57.31 km away from the	102.00 km away from the	103.29 km away from the	104.30 km away from the

(TR_0 = 4 m)	minimum tidal range	entrance for base case – it moves downstream at the entrance	entrance for base case – it moves downstream by 14% and 46% for 1 and 2 m SLR, respectively	entrance for base case – it moves downstream by 7% and 8% for 1 and 2 m SLR, respectively	entrance for base case – it moves downstream by 16% and 96% for 1 and 2 m SLR, respectively	entrance for base case – it moves downstream by 6% and 14% for 1 and 2 m SLR, respectively	entrance for base case – it moves downstream by 0.21% and 5% for 1 and 2 m SLR, respectively	entrance for base case – it moves downstream by 1% and 6% for 1 and 2 m SLR, respectively	entrance for base case – it moves downstream by 4% and 8% for 1 and 2 m SLR, respectively	entrance for base case – it moves upstream by 7% and 11% for 1 and 2 m SLR, respectively
	Tidal range pattern	X1 but SLR takes cases to A	X2 but SLR of 2m takes cases to X1	X2	X2 but SLR of 2m takes cases to X1	X2	X2	X2	X2	X2
