# Supplementary material for: Estuarine tidal range dynamics under rising sea levels
Source: PLoS One. 2021 Sep 20;16(9):e0257538. doi: 10.1371/journal.pone.0257538 (PMC8452028; doi:10.1371/journal.pone.0257538)
Supplement: S2 Table — (PDF) [file pone.0257538.s002.pdf]

S2 Table. A summary of estuarine tidal range responses to SLR during low river discharge conditions (Q/TP = 1%) for prismatic estuaries.

		Short estuary (Z = 40 km)			Moderate estuary (Z = 80 km)			Long estuary (Z = 160 km)		
**Initial tidal range**	**Tidal range response**	**Low friction (n = 0.015 s/m$^{1/3}$)**	**Mod friction (n = 0.03 s/m$^{1/3}$)**	**High friction (n = 0.09 s/m$^{1/3}$)**	**Low friction (n = 0.015 s/m$^{1/3}$)**	**Mod friction (n = 0.03 s/m$^{1/3}$)**	**High friction (n = 0.09 s/m$^{1/3}$)**	**Low friction (n = 0.015 s/m$^{1/3}$)**	**Mod friction (n = 0.03 s/m$^{1/3}$)**	**High friction (n = 0.09 s/m$^{1/3}$)**
Low (TR_0 = 0.5 m)	Location of minimum tidal range	Entrance	Entrance	23.48 km away from the entrance for base case – it moves downstream by 9% and 44% for 1 and 2 m SLR, respectively	13.55 km away from the entrance for base case – it moves downstream by 58% and 100% for 1 and 2 m SLR, respectively	23.39 km away from the entrance for base case – it moves downstream by 29% and 58% for 1 and 2 m SLR, respectively	38.64 km away from the entrance for base case – it moves upstream by 10% and 39% for 1 and 2 m SLR, respectively	84.06 km away from the entrance for base case – it moves downstream by 10% and 20% for 1 and 2 m SLR, respectively	95.68 km away from the entrance for base case – it moves downstream by 14% and 21% for 1 and 2 m SLR, respectively	37.57 km away from the entrance for base case – it moves upstream by 151% and 222% for 1 and 2 m SLR, respectively
	Tidal range pattern	A	A	X2	X1 but SLR of 2m takes cases to A	X2 but SLR takes cases to X1	D1 but SLR takes cases to X2	X2 but SLR of 2m takes cases to X1	X2	D1 but SLR takes cases to X2
Medium (TR_0 = 1 m)	Location of minimum tidal range	Entrance	5.74 km away from the entrance for base case – it moves downstream at the entrance	20.45 km away from the entrance for base case – it moves upstream by 0.90% and 0.99% for 1 and 2 m SLR, respectively	19.77 km away from the entrance for base case – it moves downstream by 39% and 58% for 1 and 2 m SLR, respectively	28.21 km away from the entrance for base case – it moves downstream by 1% and 28% for 1 and 2 m SLR, respectively	29.00 km away from the entrance for base case – it moves upstream by 87% and 102% for 1 and 2 m SLR, respectively	91.47 km away from the entrance for base case – it moves downstream by 13% and 25% for 1 and 2 m SLR, respectively	102.09 km away from the entrance for base case – it moves downstream by 5% and 13% decrease for 1 and 2 m SLR, respectively	28.55 km away from the entrance for base case – it moves upstream by 20% and 338% for 1 and 2 m SLR, respectively
	Tidal range pattern	A	X1 but SLR takes cases to A	D1 but SLR of 2m takes cases to X2	X1	X2	D1 but SLR takes cases to X2	X2	X2	D1 but SLR of 2m takes cases to X2
High	Location of	6.75 km away from the	14.21 km away from the	9.97 km away from the	31.22 km away from the	39.65 km away from the	11.54 km away from the	106.50 km away from the	113.11 km away from the	11.52 km away from the

(TR_0 = 4 m)	minimum tidal range	entrance for base case – it moves downstream at the entrance	entrance for base case – it moves downstream by 21% and 35% for 1 and 2 m SLR, respectively	entrance for base case – it moves upstream by 18% and 36% for 1 and 2 m SLR, respectively	entrance for base case – it moves downstream by 16% and 27% for 1 and 2 m SLR, respectively	entrance for base case – it moves upstream by 6% and 10% for 1 and 2 m SLR, respectively	entrance for base case – it moves upstream by 33% and 455% for 1 and 2 m SLR, respectively	entrance for base case – it moves downstream by 5% and 10% for 1 and 2 m SLR, respectively	entrance for base case – it moves downstream by 7% and 13% for 1 and 2 m SLR, respectively	entrance for base case – it moves upstream by 31% and 70% for 1 and 2 m SLR, respectively
	Tidal range pattern	X1 but SLR takes cases to A	X2 but SLR of 2m takes cases to X1	D1	X2	X2	D1 but SLR of 2m takes cases to X2	X2	X2	D1
