# Supplementary material for: Estuarine tidal range dynamics under rising sea levels
Source: PLoS One. 2021 Sep 20;16(9):e0257538. doi: 10.1371/journal.pone.0257538 (PMC8452028; doi:10.1371/journal.pone.0257538)
Supplement: S3 Table — (PDF) [file pone.0257538.s003.pdf]

S3 Table. A summary of estuarine tidal range responses to SLR during medium river discharge conditions (Q/TP = 5%) for prismatic estuaries.

		Short estuary (*Z* = 40 km)			Moderate estuary (*Z* = 80 km)			Long estuary (*Z* = 160 km)		
**Initial tidal range**	**Tidal range response**	**Low friction (*n* = 0.015 s/m$^{1/3}$)**	**Mod friction (*n* = 0.03 s/m$^{1/3}$)**	**High friction (*n* = 0.09 s/m$^{1/3}$)**	**Low friction (*n* = 0.015 s/m$^{1/3}$)**	**Mod friction (*n* = 0.03 s/m$^{1/3}$)**	**High friction (*n* = 0.09 s/m$^{1/3}$)**	**Low friction (*n* = 0.015 s/m$^{1/3}$)**	**Mod friction (*n* = 0.03 s/m$^{1/3}$)**	**High friction (*n* = 0.09 s/m$^{1/3}$)**
Low (TR_0 = 0.5 m)	Location of minimum tidal range	Entrance	17.63 km away from the entrance for base case – it moves downstream at the entrance	14.41 km away from the entrance for base case – it moves upstream by 35% and 55% for 1 and 2 m SLR, respectively	19.57 km away from the entrance for base case – it moves downstream by 50% and 75% for 1 and 2 m SLR, respectively	42.66 km away from the entrance for base case – it moves downstream by 15% and 52% for 1 and 2 m SLR, respectively	14.95 km away from the entrance for base case – it moves upstream by 50% and 106% for 1 and 2 m SLR, respectively	84.26 km away from the entrance for base case – it moves downstream by 15% and 21% for 1 and 2 m SLR, respectively	75.04 km away from the entrance for base case – it moves upstream by 39% and 30% for 1 and 2 m SLR, respectively	18.53 km away from the entrance for base case – it moves upstream by 42% and 91% for 1 and 2 m SLR, respectively
	Tidal range pattern	X3 but SLR takes cases to A	D1 but SLR takes cases to A	D1	X1	D1 but SLR takes cases to X2	D1	X2	D1 but SLR takes cases to X2	D1
Medium (TR_0 = 1 m)	Location of minimum tidal range	Entrance	24.48 km away from the entrance for base case – it moves downstream by 10% and 100% for 1 and 2 m SLR, respectively	8.77 km away from the entrance for base case – it moves upstream by 53% and 103% for 1 and 2 m SLR, respectively	27.00 km away from the entrance for base case – it moves downstream by 43% and 77% for 1 and 2 m SLR, respectively	38.85 km away from the entrance for base case – it moves upstream by 17% and downstream by 29% for 1 and 2 m SLR, respectively	10.54 km away from the entrance for base case – it moves upstream by 55% and 118% for 1 and 2 m SLR, respectively	115.16 km away from the entrance for base case – it moves downstream by 17% and 35% for 1 and 2 m SLR, respectively	45.39 km away from the entrance for base case – it moves upstream by 57% and 147% for 1 and 2 m SLR, respectively	11.52 km away from the entrance for base case – it moves upstream by 49% and 106% for 1 and 2 m SLR, respectively
	Tidal range pattern	X3	D1 but SLR of 2m takes cases to X3	D1	X2 but SLR takes cases to X1	D1 but SLR takes cases to X2	D1	X2	D1 but SLR of 2m takes cases to X2	D1

High (TR_0 = 4 m)	Location of minimum tidal range	9.57 km away from the entrance for base case – it moves downstream at the entrance	10.38 km away from the entrance for base case – it moves upstream by 37% and 107% for 1 and 2 m SLR, respectively	3.53 km away from the entrance for base case – it moves upstream by 46% and 109% for 1 and 2 m SLR, respectively	39.25 km away from the entrance for base case – it moves downstream by 30% and 48% for 1 and 2 m SLR, respectively	12.95 km away from the entrance for base case – it moves upstream by 56% and 259% for 1 and 2 m SLR, respectively	4.32 km away from the entrance for base case – it moves upstream by 46% and 112% for 1 and 2 m SLR, respectively	121.73 km away from the entrance for base case – it moves downstream by 7% and 10% for 1 and 2 m SLR, respectively	12.73 km away from the entrance for base case – it moves upstream by 68% and 154% for 1 and 2 m SLR, respectively	4.31 km away from the entrance for base case – it moves upstream by 47% and 107% for 1 and 2 m SLR, respectively
	Tidal range pattern	D1 but SLR takes cases to X3	D1	D1	X2	D1 but SLR of 2m takes cases to X2	D1	X2	D1	D1
