# Supplementary material for: Estuarine tidal range dynamics under rising sea levels
Source: PLoS One. 2021 Sep 20;16(9):e0257538. doi: 10.1371/journal.pone.0257538 (PMC8452028; doi:10.1371/journal.pone.0257538)
Supplement: S4 Table — (PDF) [file pone.0257538.s004.pdf]

S4 Table. A summary of estuarine tidal range responses to SLR during high river discharge conditions (Q/TP = 10%) for prismatic estuaries.

		**Short estuary (Z = 40 km)**			**Moderate estuary (Z = 80 km)**			**Long estuary (Z = 160 km)**		
**Initial tidal range**	**Tidal range response**	**Low friction (n = 0.015 s/m$^{1/3}$)**	**Mod friction (n = 0.03 s/m$^{1/3}$)**	**High friction (n = 0.09 s/m$^{1/3}$)**	**Low friction (n = 0.015 s/m$^{1/3}$)**	**Mod friction (n = 0.03 s/m$^{1/3}$)**	**High friction (n = 0.09 s/m$^{1/3}$)**	**Low friction (n = 0.015 s/m$^{1/3}$)**	**Mod friction (n = 0.03 s/m$^{1/3}$)**	**High friction (n = 0.09 s/m$^{1/3}$)**
Low (TR_0 = 0.5 m)	Location of minimum tidal range	Entrance	23.68 km away from the entrance for base case – it moves downstream by 21% and 26% for 1 and 2 m SLR, respectively	7.56 km away from the entrance for base case – it moves upstream by 59% and 112% for 1 and 2 m SLR, respectively	34.63 km away from the entrance for base case – it moves downstream by 48% and 59% for 1 and 2 m SLR, respectively	32.42 km away from the entrance for base case – it moves upstream by 23% and 25% for 1 and 2 m SLR, respectively	7.93 km away from the entrance for base case – it moves upstream by 61% and 129% for 1 and 2 m SLR, respectively	97.28 km away from the entrance for base case – it moves downstream by 12% and 16% for 1 and 2 m SLR, respectively	40.17 km away from the entrance for base case – it moves upstream by 57% and 117% for 1 and 2 m SLR, respectively	10.72 km away from the entrance for base case – it moves upstream by 49% and 107% for 1 and 2 m SLR, respectively
	Tidal range pattern	X3	D1	D1	X2 but SLR takes cases to X1	D1	D1	X2	D1	D1
Medium (TR_0 = 1 m)	Location of minimum tidal range	12.00 km away from the entrance for base case – it moves upstream by 12% and downstream by 100% for 1 and 2 m SLR, respectively	16.02 km away from the entrance for base case – it moves upstream by 58% and 75% for 1 and 2 m SLR, respectively	4.13 km away from the entrance for base case – it moves upstream by 59% and 141% for 1 and 2 m SLR, respectively	43.46 km away from the entrance for base case – it moves downstream by 32% and 66% for 1 and 2 m SLR, respectively	22.58 km away from the entrance for base case – it moves upstream by 47% and 74% for 1 and 2 m SLR, respectively	5.12 km away from the entrance for base case – it moves upstream by 63% and 145% for 1 and 2 m SLR, respectively	109.71 km away from the entrance for base case – it moves downstream by 2% and 12% for 1 and 2 m SLR, respectively	24.14 km away from the entrance for base case – it moves upstream by 58% and 134% for 1 and 2 m SLR, respectively	5.51 km away from the entrance for base case – it moves upstream by 62% and 142% for 1 and 2 m SLR, respectively
	Tidal range pattern	D1 but SLR of 1m and 2m take cases to	D1	D1	X2 but SLR of 2m takes cases to X1	D1	D1	X2	D1	D1

		X2 and X3, respectively								
High (TR_0 = 4 m)	Location of minimum tidal range	10.18 km away from the entrance for base case – it moves upstream by 57% and 116% for 1 and 2 m SLR, respectively	5.34 km away from the entrance for base case – it moves upstream by 53% and 140% for 1 and 2 m SLR, respectively	1.91 km away from the entrance for base case – it moves upstream by 32% and 84% for 1 and 2 m SLR, respectively	11.34 km away from the entrance for base case – it moves upstream by 69% and 281% for 1 and 2 m SLR, respectively	6.73 km away from the entrance for base case – it moves upstream by 57% and 143% for 1 and 2 m SLR, respectively	2.31 km away from the entrance for base case – it moves upstream by 35% and 87% for 1 and 2 m SLR, respectively	12.92 km away from the entrance for base case – it moves upstream by 70% and 86% for 1 and 2 m SLR, respectively	6.51 km away from the entrance for base case – it moves upstream by 62% and 160% for 1 and 2 m SLR, respectively	2.30 km away from the entrance for base case – it moves upstream by 35% and 87% for 1 and 2 m SLR, respectively
	Tidal range pattern	D1	D1	D1	D1 but SLR takes cases to X2	D1	D1	D1 but SLR takes cases to X2	D1	D1
