# Supplementary material for: Estuarine tidal range dynamics under rising sea levels
Source: PLoS One. 2021 Sep 20;16(9):e0257538. doi: 10.1371/journal.pone.0257538 (PMC8452028; doi:10.1371/journal.pone.0257538)
Supplement: S5 Table — (PDF) [file pone.0257538.s005.pdf]

S5 Table. A summary of estuarine tidal range responses to SLR during no river discharge conditions (Q/TP = 0%) for converging estuaries with L_c = 160 km.

		Short estuary (*Z* = 40 km)			Moderate estuary (*Z* = 80 km)			Long estuary (*Z* = 160 km)		
**Initial tidal range**	**Tidal range response**	**Low friction (*n* = 0.015 s/m$^{1/3}$)**	**Mod friction (*n* = 0.03 s/m$^{1/3}$)**	**High friction (*n* = 0.09 s/m$^{1/3}$)**	**Low friction (*n* = 0.015 s/m$^{1/3}$)**	**Mod friction (*n* = 0.03 s/m$^{1/3}$)**	**High friction (*n* = 0.09 s/m$^{1/3}$)**	**Low friction (*n* = 0.015 s/m$^{1/3}$)**	**Mod friction (*n* = 0.03 s/m$^{1/3}$)**	**High friction (*n* = 0.09 s/m$^{1/3}$)**
Low (TR_0 = 0.5 m)	Location of minimum tidal range	Entrance	Entrance	14.7 km away from the entrance for base case – it moves downstream by 23% and 36% for 1 and 2 m SLR, respectively	8.38 km away from the entrance for base case – it moves downstream at the entrance	19.88 km away from the entrance for base case – it moves downstream by 40% and 73% for 1 and 2 m SLR, respectively	45.13 km away from the entrance for base case – it moves downstream by 2% and 20% for 1 and 2 m SLR, respectively	76.63 km away from the entrance for base case – it moves downstream by 14% and 28% for 1 and 2 m SLR, respectively	84.25 km away from the entrance for base case – it moves downstream by 8% and 14% for 1 and 2 m SLR, respectively	89.00 km away from the entrance for base case – it moves upstream by 23% and 35% for 1 and 2 m SLR, respectively
	Tidal range pattern	A	A	X2 but SLR of 2m takes cases to X1	X1 but SLR takes cases to A	X1	X2	X1	X2 but SLR of 2m takes cases to X1	X2
Medium (TR_0 = 1 m)	Location of minimum tidal range	Entrance	4.65 km away from the entrance for base case – it moves downstream at the entrance	22.40 km away from the entrance for base case – it moves downstream by 17% and 34% for 1 and 2 m SLR, respectively	16.63 km away from the entrance for base case – it moves downstream by 57% and 100% for 1 and 2 m SLR, respectively	35.38 km away from the entrance for base case – it moves downstream by 21% and 42% for 1 and 2 m SLR, respectively	52.63 km away from the entrance for base case – it moves downstream by 9% and 12% for 1 and 2 m SLR, respectively	84.25 km away from the entrance for base case – it moves downstream by 9% and 25% for 1 and 2 m SLR, respectively	96.13 km away from the entrance for base case – it moves downstream by 12% and 17% for 1 and 2 m SLR, respectively	108.12 km away from the entrance for base case – it moves upstream by 4% and 8% for 1 and 2 m SLR, respectively
	Tidal range pattern	A	X1 but SLR takes cases to A	X2	X1 but SLR of 2m takes cases to A	X2 but SLR of 2m takes cases to X1	X2	X2 but SLR takes cases to X1	X2	X2
High	Location of	Entrance	14.65 km away from the	27.95 km away from the	32.13 km away from the	46.50 km away from the	56.63 km away from the	108.75 km away from the	107.13 km away from the	109.00 km away from the

(TR_0 = 4 m)	minimum tidal range		entrance for base case – it moves downstream by 28% and 60% for 1 and 2 m SLR, respectively	entrance for base case – it moves downstream by 6% and 18% for 1 and 2 m SLR, respectively	entrance for base case – it moves downstream by 13% and 34% for 1 and 2 m SLR, respectively	entrance for base case – it moves downstream by 7% and 16% for 1 and 2 m SLR, respectively	entrance for base case – it moves downstream by 0.2% and 3% for 1 and 2 m SLR, respectively	entrance for base case – it moves downstream by 5% and 10% for 1 and 2 m SLR, respectively	entrance for base case – it moves downstream by 4% and 10% for 1 and 2 m SLR, respectively	entrance for base case – it moves upstream by 6% and 12% for 1 and 2 m SLR, respectively
	Tidal range pattern	A	X2 but SLR of 2m takes cases to X1	X2	X2 but SLR of 2m takes cases to X1	X2	X2	X2	X2	X2
