# Supplementary material for: Estuarine tidal range dynamics under rising sea levels
Source: PLoS One. 2021 Sep 20;16(9):e0257538. doi: 10.1371/journal.pone.0257538 (PMC8452028; doi:10.1371/journal.pone.0257538)
Supplement: S6 Table — (PDF) [file pone.0257538.s006.pdf]

S6 Table. A summary of estuarine tidal range responses to SLR during low river discharge conditions (Q/TP = 1%) for converging estuaries with L_c = 160 km.

		**Short estuary (*Z* = 40 km)**			**Moderate estuary (*Z* = 80 km)**			**Long estuary (*Z* = 160 km)**		
**Initial tidal range**	**Tidal range response**	**Low friction (*n* = 0.015 s/m$^{1/3}$)**	**Mod friction (*n* = 0.03 s/m$^{1/3}$)**	**High friction (*n* = 0.09 s/m$^{1/3}$)**	**Low friction (*n* = 0.015 s/m$^{1/3}$)**	**Mod friction (*n* = 0.03 s/m$^{1/3}$)**	**High friction (*n* = 0.09 s/m$^{1/3}$)**	**Low friction (*n* = 0.015 s/m$^{1/3}$)**	**Mod friction (*n* = 0.03 s/m$^{1/3}$)**	**High friction (*n* = 0.09 s/m$^{1/3}$)**
Low (TR_0 = 0.5 m)	Location of minimum tidal range	Entrance	Entrance	19.25 km away from the entrance for base case – it moves downstream by 17% and 38% for 1 and 2 m SLR, respectively	8.63 km away from the entrance for base case – it moves downstream at the entrance	22.50 km away from the entrance for base case – it moves downstream by 45% and 76% for 1 and 2 m SLR, respectively	43.12 km away from the entrance for base case – it moves downstream by 4% and upstream by 19% for 1 and 2 m SLR, respectively	76.25 km away from the entrance for base case – it moves downstream by 15% and 31% for 1 and 2 m SLR, respectively	100.88 km away from the entrance for base case – it moves downstream by 18% and 27% for 1 and 2 m SLR, respectively	54.63 km away from the entrance for base case – it moves upstream by 35% and 62% for 1 and 2 m SLR, respectively
	Tidal range pattern	A	A	X2 but SLR of 2m takes cases to X1	X1 but SLR takes cases to A	X2 but SLR takes cases to X1	D1 but SLR of 2m takes cases to X2	X1	X2	D1
Medium (TR_0 = 1 m)	Location of minimum tidal range	Entrance	4.40 km away from the entrance for base case – it moves downstream at the entrance	22.25 km away from the entrance for base case – it moves downstream by 9% and 1% for 1 and 2 m SLR, respectively	17.62 km away from the entrance for base case – it moves downstream by 57% and 100% for 1 and 2 m SLR, respectively	27.63 km away from the entrance for base case – it moves downstream by 15% and 43% for 1 and 2 m SLR, respectively	37.13 km away from the entrance for base case – it moves upstream by 10% and 66% for 1 and 2 m SLR, respectively	89.12 km away from the entrance for base case – it moves downstream by 15% and 33% for 1 and 2 m SLR, respectively	107.50 km away from the entrance for base case – it moves downstream by 9% and 17% for 1 and 2 m SLR, respectively	40.13 km away from the entrance for base case – it moves upstream by 34% and 72% for 1 and 2 m SLR, respectively
	Tidal range pattern	A	X1 but SLR takes cases to A	D1 but SLR of 2m takes cases to X2	X1 but SLR of 2m takes cases to A	X2 but SLR of 2m takes cases to X1	D1 but SLR of 2m takes cases to X2	X2 but SLR takes cases to X1	X2	D1

High (TR_0 = 4 m)	Location of minimum tidal range	Entrance	15.65 km away from the entrance for base case – it moves downstream by 22% and 50% for 1 and 2 m SLR, respectively	17.70 km away from the entrance for base case – it moves upstream by 11% and 13% for 1 and 2 m SLR, respectively	30.00 km away from the entrance for base case – it moves downstream by 18% and 35% for 1 and 2 m SLR, respectively	36.62 km away from the entrance for base case – it moves upstream by 19% and 60% for 1 and 2 m SLR, respectively	24.38 km away from the entrance for base case – it moves upstream by 22% and 35% for 1 and 2 m SLR, respectively	108.50 km away from the entrance for base case – it moves downstream by 6% and 11% for 1 and 2 m SLR, respectively	126.00 km away from the entrance for base case – it moves downstream by 13% and 19% for 1 and 2 m SLR, respectively	24.50 km away from the entrance for base case – it moves upstream by 28% and 58% for 1 and 2 m SLR, respectively
	Tidal range pattern	A	X2 but SLR of 2m takes cases to X1	D1	X2 but SLR of 2m takes cases to X1	X2	D1	X2	X2	D1
