# Supplementary material for: Estuarine tidal range dynamics under rising sea levels
Source: PLoS One. 2021 Sep 20;16(9):e0257538. doi: 10.1371/journal.pone.0257538 (PMC8452028; doi:10.1371/journal.pone.0257538)
Supplement: S7 Table — (PDF) [file pone.0257538.s007.pdf]

S7 Table. A summary of estuarine tidal range responses to SLR during medium river discharge conditions (Q/TP = 5%) for converging estuaries with L_c = 160 km.

		Short estuary (*Z* = 40 km)			Moderate estuary (*Z* = 80 km)			Long estuary (*Z* = 160 km)		
**Initial tidal range**	**Tidal range response**	**Low friction (*n* = 0.015 s/m$^{1/3}$)**	**Mod friction (*n* = 0.03 s/m$^{1/3}$)**	**High friction (*n* = 0.09 s/m$^{1/3}$)**	**Low friction (*n* = 0.015 s/m$^{1/3}$)**	**Mod friction (*n* = 0.03 s/m$^{1/3}$)**	**High friction (*n* = 0.09 s/m$^{1/3}$)**	**Low friction (*n* = 0.015 s/m$^{1/3}$)**	**Mod friction (*n* = 0.03 s/m$^{1/3}$)**	**High friction (*n* = 0.09 s/m$^{1/3}$)**
Low (TR_0 = 0.5 m)	Location of minimum tidal range	Entrance	37.45 km away from the entrance for base case – it moves downstream at the entrance	16.40 km away from the entrance for base case – it moves upstream by 25% and 45% for 1 and 2 m SLR, respectively	13.13 km away from the entrance for base case – it moves downstream by 90% and 100% for 1 and 2 m SLR, respectively	44.75 km away from the entrance for base case – it moves downstream by 11% and 54% for 1 and 2 m SLR, respectively	18.00 km away from the entrance for base case – it moves upstream by 42% and 85% for 1 and 2 m SLR, respectively	104.50 km away from the entrance for base case – it moves downstream by 33% and 48% for 1 and 2 m SLR, respectively	78.00 km away from the entrance for base case – it moves upstream by 27% and 47% for 1 and 2 m SLR, respectively	20.88 km away from the entrance for base case – it moves upstream by 43% and 89% for 1 and 2 m SLR, respectively
	Tidal range pattern	A	D1 but SLR of 1m and 2m take cases to X3 and A, respectively	D1	X1 but SLR of 2m takes cases to A	D1 but SLR of 1m and 2m take cases to X2 and X1, respectively	D1	X2 but SLR takes cases to X1	D1 but SLR of 2m takes cases to X2	D1
Medium (TR_0 = 1 m)	Location of minimum tidal range	Entrance	32.35 km away from the entrance for base case – it moves upstream by 15% and downstream by 71% for 1 and 2 m SLR, respectively	11.50 km away from the entrance for base case – it moves upstream by 38% and 73% for 1 and 2 m SLR, respectively	26.00 km away from the entrance for base case – it moves downstream by 55% and 100% for 1 and 2 m SLR, respectively	41.38 km away from the entrance for base case – it moves upstream by 11% and downstream by 8% for 1 and 2 m SLR, respectively	13.62 km away from the entrance for base case – it moves upstream by 45% and 95% for 1 and 2 m SLR, respectively	118.50 km away from the entrance for base case – it moves downstream by 11% and 38% for 1 and 2 m SLR, respectively	54.75 km away from the entrance for base case – it moves upstream by 45% and 78% for 1 and 2 m SLR, respectively	14.50 km away from the entrance for base case – it moves upstream by 44% and 94% for 1 and 2 m SLR, respectively

	Tidal range pattern	X3	D1 but SLR of 2m takes cases to X2	D1	X2 but SLR of 1m and 2m take cases to X1 and X3, respectively	D1 but SLR of 2m takes cases to X2	D1	X2 but SLR of 2m takes cases to X1	D1	D1
High (TR_0 = 4 m)	Location of minimum tidal range	34.05 km away from the entrance for base case – it moves upstream by 11% and 15% for 1 and 2 m SLR, respectively	20.90 km away from the entrance for base case – it moves upstream by 39% and 64% for 1 and 2 m SLR, respectively	5.25 km away from the entrance for base case – it moves upstream by 52% and 114% for 1 and 2 m SLR, respectively	39.63 km away from the entrance for base case – it moves downstream by 34% and 50% for 1 and 2 m SLR, respectively	27.37 km away from the entrance for base case – it moves upstream by 37% and 60% for 1 and 2 m SLR, respectively	7.12 km away from the entrance for base case – it moves upstream by 47% and 104% for 1 and 2 m SLR, respectively	68.50 km away from the entrance for base case – it moves upstream by 80% and 72% for 1 and 2 m SLR, respectively	29.25 km away from the entrance for base case – it moves upstream by 51% and 113% for 1 and 2 m SLR, respectively	7.00 km away from the entrance for base case – it moves upstream by 48% and 105% for 1 and 2 m SLR, respectively
	Tidal range pattern	D1	D1	D1	X2	D1	D1	D1 but SLR takes cases to X2	D1	D1
