# Supplementary material for: Estuarine tidal range dynamics under rising sea levels
Source: PLoS One. 2021 Sep 20;16(9):e0257538. doi: 10.1371/journal.pone.0257538 (PMC8452028; doi:10.1371/journal.pone.0257538)
Supplement: S8 Table — (PDF) [file pone.0257538.s008.pdf]

S8 Table. A summary of estuarine tidal range responses to SLR during high river discharge conditions (Q/TP = 10%) for converging estuaries with L_c = 160 km.

		Short estuary (*Z* = 40 km)			Moderate estuary (*Z* = 80 km)			Long estuary (*Z* = 160 km)		
**Initial tidal range**	**Tidal range response**	**Low friction (*n* = 0.015 s/m$^{1/3}$)**	**Mod friction (*n* = 0.03 s/m$^{1/3}$)**	**High friction (*n* = 0.09 s/m$^{1/3}$)**	**Low friction (*n* = 0.015 s/m$^{1/3}$)**	**Mod friction (*n* = 0.03 s/m$^{1/3}$)**	**High friction (*n* = 0.09 s/m$^{1/3}$)**	**Low friction (*n* = 0.015 s/m$^{1/3}$)**	**Mod friction (*n* = 0.03 s/m$^{1/3}$)**	**High friction (*n* = 0.09 s/m$^{1/3}$)**
Low (TR_0 = 0.5 m)	Location of minimum tidal range	Entrance	30.60 km away from the entrance for base case – it moves upstream by 18% and 26% for 1 and 2 m SLR, respectively	10.45 km away from the entrance for base case – it moves upstream by 41% and 79% for 1 and 2 m SLR, respectively	43.25 km away from the entrance for base case – it moves downstream by 61% and 100% for 1 and 2 m SLR, respectively	36.00 km away from the entrance for base case – it moves upstream by 24% and 43% for 1 and 2 m SLR, respectively	10.88 km away from the entrance for base case – it moves upstream by 48% and 102% for 1 and 2 m SLR, respectively	95.75 km away from the entrance for base case – it moves upstream by 7% and downstream by 13% for 1 and 2 m SLR, respectively	47.75 km away from the entrance for base case – it moves upstream by 45% and 85% for 1 and 2 m SLR, respectively	13.00 km away from the entrance for base case – it moves upstream by 44% and 96% for 1 and 2 m SLR, respectively
	Tidal range pattern	X3	D1	D1	X2 but SLR of 2m takes cases to X3	D1	D1	D1 but SLR takes cases to X2	D1	D1
Medium (TR_0 = 1 m)	Location of minimum tidal range	37.10 km away from the entrance for base case – it moves upstream by 6% and downstream by 100% for 1 and 2 m SLR, respectively	22.90 km away from the entrance for base case – it moves upstream by 35% and 54% for 1 and 2 m SLR, respectively	6.20 km away from the entrance for base case – it moves upstream by 54% and 113% for 1 and 2 m SLR, respectively	53.63 km away from the entrance for base case – it moves downstream by 33% and 76% for 1 and 2 m SLR, respectively	28.25 km away from the entrance for base case – it moves upstream by 35% and 67% for 1 and 2 m SLR, respectively	7.63 km away from the entrance for base case – it moves upstream by 52% and 115% for 1 and 2 m SLR, respectively	76.25 km away from the entrance for base case – it moves upstream by 26% and 44% for 1 and 2 m SLR, respectively	32.00 km away from the entrance for base case – it moves upstream by 48% and 104% for 1 and 2 m SLR, respectively	8.25 km away from the entrance for base case – it moves upstream by 50% and 111% for 1 and 2 m SLR, respectively
	Tidal range pattern	D1 but SLR of 1m and 2m take cases to	D1	D1	D1 but SLR of 1m and 2m take cases to	D1	D1	D1 but SLR of 2m takes cases to X2	D1	D1

		D2 and X3, respectively			X2 and X1, respectively					
High (TR_0 = 4 m)	Location of minimum tidal range	20.35 km away from the entrance for base case – it moves upstream by 43% and 67% for 1 and 2 m SLR, respectively	10.25 km away from the entrance for base case – it moves upstream by 60% and 130% for 1 and 2 m SLR, respectively	2.20 km away from the entrance for base case – it moves upstream by 64% and 148% for 1 and 2 m SLR, respectively	32.63 km away from the entrance for base case – it moves upstream by 39% and 103% for 1 and 2 m SLR, respectively	13.88 km away from the entrance for base case – it moves upstream by 51% and 109% for 1 and 2 m SLR, respectively	3.38 km away from the entrance for base case – it moves upstream by 52% and 122% for 1 and 2 m SLR, respectively	31.25 km away from the entrance for base case – it moves upstream by 84% and 116% for 1 and 2 m SLR, respectively	14.00 km away from the entrance for base case – it moves upstream by 56% and 126% for 1 and 2 m SLR, respectively	3.38 km away from the entrance for base case – it moves upstream by 52% and 119% for 1 and 2 m SLR, respectively
	Tidal range pattern	D1	D1	D1	D1	D1	D1	D1	D1	D1
