# Supplementary material for: Estuarine tidal range dynamics under rising sea levels
Source: PLoS One. 2021 Sep 20;16(9):e0257538. doi: 10.1371/journal.pone.0257538 (PMC8452028; doi:10.1371/journal.pone.0257538)
Supplement: S9 Table — (PDF) [file pone.0257538.s009.pdf]

S9 Table. A summary of estuarine tidal range responses to SLR during no river discharge conditions (Q/TP = 0%) for converging estuaries with L_c = 80 km.

		**Short estuary (*Z* = 40 km)**			**Moderate estuary (*Z* = 80 km)**			**Long estuary (*Z* = 160 km)**		
**Initial tidal range**	**Tidal range response**	**Low friction (*n* = 0.015 s/m$^{1/3}$)**	**Mod friction (*n* = 0.03 s/m$^{1/3}$)**	**High friction (*n* = 0.09 s/m$^{1/3}$)**	**Low friction (*n* = 0.015 s/m$^{1/3}$)**	**Mod friction (*n* = 0.03 s/m$^{1/3}$)**	**High friction (*n* = 0.09 s/m$^{1/3}$)**	**Low friction (*n* = 0.015 s/m$^{1/3}$)**	**Mod friction (*n* = 0.03 s/m$^{1/3}$)**	**High friction (*n* = 0.09 s/m$^{1/3}$)**
Low (TR_0 = 0.5 m)	Location of minimum tidal range	Entrance	Entrance	15.20 km away from the entrance for base case – it moves downstream by 26% and 48% for 1 and 2 m SLR, respectively	Entrance	16.75 km away from the entrance for base case – it moves downstream by 60% and 100% for 1 and 2 m SLR, respectively	46.88 km away from the entrance for base case – it moves downstream by 14% and 19% for 1 and 2 m SLR, respectively	69.75 km away from the entrance for base case – it moves downstream by 31% and 58% for 1 and 2 m SLR, respectively	82.62 km away from the entrance for base case – it moves downstream by 4% and 19% for 1 and 2 m SLR, respectively	92.25 km away from the entrance for base case – it moves upstream by 3% and 5% for 1 and 2 m SLR, respectively
	Tidal range pattern	A	A	X2 but SLR of 2m takes cases to X1	A	X1 but SLR of 2m takes cases to A	X2	X1	X2 but SLR takes cases to X1	X2
Medium (TR_0 = 1 m)	Location of minimum tidal range	Entrance	1.9 km away from the entrance for base case – it moves downstream at the entrance	20.20 km away from the entrance for base case – it moves downstream by 16% and 28% for 1 and 2 m SLR, respectively	10.75 km away from the entrance for base case – it moves downstream at the entrance	33.25 km away from the entrance for base case – it moves downstream by 29% and 68% for 1 and 2 m SLR, respectively	54.50 km away from the entrance for base case – it moves downstream by 6% and 13% for 1 and 2 m SLR, respectively	76.12 km away from the entrance for base case – it moves downstream by 17% and 41% for 1 and 2 m SLR, respectively	93.75 km away from the entrance for base case – it moves downstream by 10% and 15% decrease for 1 and 2 m SLR, respectively	111.38 km away from the entrance for base case – it moves upstream by 1% and 2% for 1 and 2 m SLR, respectively
	Tidal range pattern	A	X1 but SLR takes cases to A	X2	X1 but SLR takes cases to A	X2 but SLR takes cases to X1	X2	X1	X2 but SLR of 2m takes cases to X1	X2

High (TR_0 = 4 m)	Location of minimum tidal range	4.25 km away from the entrance for base case – it moves downstream at the entrance	13.30 km away from the entrance for base case – it moves downstream by 32% and 48% for 1 and 2 m SLR, respectively	25.55 km away from the entrance for base case – it moves downstream by 2% and 6% for 1 and 2 m SLR, respectively	32.62 km away from the entrance for base case – it moves downstream by 16% and 45% for 1 and 2 m SLR, respectively	48.13 km away from the entrance for base case – it moves downstream by 16% and 23% for 1 and 2 m SLR, respectively	57.50 km away from the entrance for base case – it moves downstream by 0.2% and 3% for 1 and 2 m SLR, respectively	107.63 km away from the entrance for base case – it moves downstream by 7% and 9% for 1 and 2 m SLR, respectively	109.50 km away from the entrance for base case – it moves downstream by 4% and 10% for 1 and 2 m SLR, respectively	113.50 km away from the entrance for base case – it moves upstream by 0.2% and 7% for 1 and 2 m SLR, respectively
	Tidal range pattern	X1 but SLR takes cases to A	X2 but SLR takes cases to X1	X2	X1	X2	X2	X2	X2	X2
