# Supplementary material for: Estuarine tidal range dynamics under rising sea levels
Source: PLoS One. 2021 Sep 20;16(9):e0257538. doi: 10.1371/journal.pone.0257538 (PMC8452028; doi:10.1371/journal.pone.0257538)
Supplement: S10 Table — (PDF) [file pone.0257538.s010.pdf]

S10 Table. A summary of estuarine tidal range responses to SLR during low river discharge conditions (Q/TP = 1%) for converging estuaries with L_c = 80 km.

		Short estuary (*Z* = 40 km)			Moderate estuary (*Z* = 80 km)			Long estuary (*Z* = 160 km)		
**Initial tidal range**	**Tidal range response**	**Low friction (*n* = 0.015 s/m$^{1/3}$)**	**Mod friction (*n* = 0.03 s/m$^{1/3}$)**	**High friction (*n* = 0.09 s/m$^{1/3}$)**	**Low friction (*n* = 0.015 s/m$^{1/3}$)**	**Mod friction (*n* = 0.03 s/m$^{1/3}$)**	**High friction (*n* = 0.09 s/m$^{1/3}$)**	**Low friction (*n* = 0.015 s/m$^{1/3}$)**	**Mod friction (*n* = 0.03 s/m$^{1/3}$)**	**High friction (*n* = 0.09 s/m$^{1/3}$)**
Low (TR_0 = 0.5 m)	Location of minimum tidal range	Entrance	Entrance	21.90 km away from the entrance for base case – it moves downstream by 31% and 66% for 1 and 2 m SLR, respectively	Entrance	19.12 km away from the entrance for base case – it moves downstream by 53% and 100% for 1 and 2 m SLR, respectively	47.00 km away from the entrance for base case – it moves upstream by 9% and 31% for 1 and 2 m SLR, respectively	60.00 km away from the entrance for base case – it moves downstream by 34% and 58% for 1 and 2 m SLR, respectively	104.00 km away from the entrance for base case – it moves downstream by 17% and 32% for 1 and 2 m SLR, respectively	56.38 km away from the entrance for base case – it moves upstream by 36% and 73% for 1 and 2 m SLR, respectively
	Tidal range pattern	A	A	D1 but SLR of 1m and 2m take cases to X2 and X1, respectively	A	X1 but SLR of 2m takes cases to A	D1 but SLR of 2m takes cases to X2	X1	X2 but SLR of 2m takes cases to X1	D1
Medium (TR_0 = 1 m)	Location of minimum tidal range	Entrance	2.15 km away from the entrance for base case – it moves downstream at the entrance	24.60 km away from the entrance for base case – it moves downstream by 19% and 39% for 1 and 2 m SLR, respectively	11.63 km away from the entrance for base case – it moves downstream at the entrance	25.75 km away from the entrance for base case – it moves downstream by 28% and 59% for 1 and 2 m SLR, respectively	39.50 km away from the entrance for base case – it moves upstream by 21% and 25% for 1 and 2 m SLR, respectively	82.62 km away from the entrance for base case – it moves downstream by 26% and 60% for 1 and 2 m SLR, respectively	115.63 km away from the entrance for base case – it moves downstream by 11% and 24% for 1 and 2 m SLR, respectively	41.75 km away from the entrance for base case – it moves upstream by 36% and 76% increase for 1 and 2 m SLR, respectively
	Tidal range pattern	A	X1 but SLR takes cases to A	D1 but SLR takes cases to X2	X1 but SLR takes cases to A	X2 but SLR takes cases to X1	D1	X1	D1 but SLR takes cases to X2	D1

High (TR_0 = 4 m)	Location of minimum tidal range	2.40 km away from the entrance for base case – it moves downstream at the entrance	13.95 km away from the entrance for base case – it moves downstream by 16% and 56% for 1 and 2 m SLR, respectively	19.65 km away from the entrance for base case – it moves upstream by 14% and 21% for 1 and 2 m SLR, respectively	29.00 km away from the entrance for base case – it moves downstream by 20% and 44% for 1 and 2 m SLR, respectively	38.12 km away from the entrance for base case – it moves downstream by 4% and 11% for 1 and 2 m SLR, respectively	26.25 km away from the entrance for base case – it moves upstream by 28% and 49% for 1 and 2 m SLR, respectively	107.63 km away from the entrance for base case – it moves downstream by 6% and 20% for 1 and 2 m SLR, respectively	87.00 km away from the entrance for base case – it moves upstream by 16% and 47% for 1 and 2 m SLR, respectively	26.25 km away from the entrance for base case – it moves upstream by 31% and 66% for 1 and 2 m SLR, respectively
	Tidal range pattern	X1 but SLR takes cases to A	X2 but SLR takes cases to X1	D1	X1	X2	D1	X2	D1 but SLR of 2m takes cases to X2	D1
