# Supplementary material for: Estuarine tidal range dynamics under rising sea levels
Source: PLoS One. 2021 Sep 20;16(9):e0257538. doi: 10.1371/journal.pone.0257538 (PMC8452028; doi:10.1371/journal.pone.0257538)
Supplement: S11 Table — (PDF) [file pone.0257538.s011.pdf]

S11 Table. A summary of estuarine tidal range responses to SLR during medium river discharge conditions (Q/TP = 5%) for converging estuaries with L_c = 80 km.

		Short estuary (*Z* = 40 km)			Moderate estuary (*Z* = 80 km)			Long estuary (*Z* = 160 km)		
**Initial tidal range**	**Tidal range response**	**Low friction (*n* = 0.015 s/m$^{1/3}$)**	**Mod friction (*n* = 0.03 s/m$^{1/3}$)**	**High friction (*n* = 0.09 s/m$^{1/3}$)**	**Low friction (*n* = 0.015 s/m$^{1/3}$)**	**Mod friction (*n* = 0.03 s/m$^{1/3}$)**	**High friction (*n* = 0.09 s/m$^{1/3}$)**	**Low friction (*n* = 0.015 s/m$^{1/3}$)**	**Mod friction (*n* = 0.03 s/m$^{1/3}$)**	**High friction (*n* = 0.09 s/m$^{1/3}$)**
Low (TR_0 = 0.5 m)	Location of minimum tidal range	Entrance	38.35 km away from the entrance for base case – it moves downstream at the entrance	17.80 km away from the entrance for base case – it moves upstream by 27% and 50% for 1 and 2 m SLR, respectively	Entrance	55.00 km away from the entrance for base case – it moves upstream by 29% and downstream by 62% for 1 and 2 m SLR, respectively	19.12 km away from the entrance for base case – it moves upstream by 42% and 87% for 1 and 2 m SLR, respectively	151.37 km away from the entrance for base case – it moves downstream at the entrance	78.62km away from the entrance for base case – it moves upstream by 35% and 63% for 1 and 2 m SLR, respectively	21.50km away from the entrance for base case – it moves upstream by 42% and 90% for 1 and 2 m SLR, respectively
	Tidal range pattern	A	D1 but SLR takes cases to X3	D1	X3 but SLR of 2m takes cases to A	D1 but SLR of 2m takes cases to X2	D1	D2 but SLR takes cases to X3	D1 but SLR takes cases to D2	D1
Medium (TR_0 = 1 m)	Location of minimum tidal range	Entrance	34.60 km away from the entrance for base case – it moves upstream by 9% and 11% for 1 and 2 m SLR, respectively	12.40 km away from the entrance for base case – it moves upstream by 39% and 76% for 1 and 2 m SLR, respectively	26.75 km away from the entrance for base case – it moves downstream at the entrance	46.62 km away from the entrance for base case – it moves upstream by 32% and 60% for 1 and 2 m SLR, respectively	14.25 km away from the entrance for base case – it moves upstream by 46% and 97% for 1 and 2 m SLR, respectively	123.25 km away from the entrance for base case – it moves upstream by 24% and downstream by 86% for 1 and 2 m SLR, respectively	56.00 km away from the entrance for base case – it moves upstream by 43% and 85% for 1 and 2 m SLR, respectively	15.00km away from the entrance for base case – it moves upstream by 44% and 95% for 1 and 2 m SLR, respectively

	Tidal range pattern	X3	D1	D1	X2 but SLR of 2m takes cases to X3	D1	D1	D2 but SLR of 2m takes cases to X2	D1	D1
High (TR_0 = 4 m)	Location of minimum tidal range	34.70 km away from the entrance for base case – it moves upstream by 9% and 11% for 1 and 2 m SLR, respectively	23.15 km away from the entrance for base case – it moves upstream by 33% and 52% for 1 and 2 m SLR, respectively	5.65 km away from the entrance for base case – it moves upstream by 53% and 116% for 1 and 2 m SLR, respectively	68.62 km away from the entrance for base case – it moves downstream by 57% and 75% for 1 and 2 m SLR, respectively	29.50 km away from the entrance for base case – it moves upstream by 40% and 86% for 1 and 2 m SLR, respectively	7.38 km away from the entrance for base case – it moves upstream by 47% and 107% for 1 and 2 m SLR, respectively	85.25 km away from the entrance for base case – it moves upstream by 16% and 44% for 1 and 2 m SLR, respectively	30.50 km away from the entrance for base case – it moves upstream by 48% and 105% for 1 and 2 m SLR, respectively	7.38 km away from the entrance for base case – it moves upstream by 47% and 105% for 1 and 2 m SLR, respectively
	Tidal range pattern	D1	D1	D1	D1 but SLR takes cases to X2	D1	D1	D1	D1	D1
