# Supplementary material for: Estuarine tidal range dynamics under rising sea levels
Source: PLoS One. 2021 Sep 20;16(9):e0257538. doi: 10.1371/journal.pone.0257538 (PMC8452028; doi:10.1371/journal.pone.0257538)
Supplement: S12 Table — (PDF) [file pone.0257538.s012.pdf]

S12 Table. A summary of estuarine tidal range responses to SLR during high river discharge conditions (Q/TP = 10%) for converging estuaries with L_c = 80 km.

		**Short estuary (*Z* = 40 km)**			**Moderate estuary (*Z* = 80 km)**			**Long estuary (*Z* = 160 km)**		
**Initial tidal range**	**Tidal range response**	**Low friction (*n* = 0.015 s/m$^{1/3}$)**	**Mod friction (*n* = 0.03 s/m$^{1/3}$)**	**High friction (*n* = 0.09 s/m$^{1/3}$)**	**Low friction (*n* = 0.015 s/m$^{1/3}$)**	**Mod friction (*n* = 0.03 s/m$^{1/3}$)**	**High friction (*n* = 0.09 s/m$^{1/3}$)**	**Low friction (*n* = 0.015 s/m$^{1/3}$)**	**Mod friction (*n* = 0.03 s/m$^{1/3}$)**	**High friction (*n* = 0.09 s/m$^{1/3}$)**
Low (TR_0 = 0.5 m)	Location of minimum tidal range	Entrance	33.25 km away from the entrance for base case – it moves upstream by 12% and 17% for 1 and 2 m SLR, respectively	11.45 km away from the entrance for base case – it moves upstream by 41% and 80% for 1 and 2 m SLR, respectively	74.87 km away from the entrance for base case – it moves downstream at the entrance	40.00 km away from the entrance for base case – it moves upstream by 33% and 67% for 1 and 2 m SLR, respectively	11.62 km away from the entrance for base case – it moves upstream by 47% and 101% for 1 and 2 m SLR, respectively	109.00 km away from the entrance for base case – it moves upstream by 31% and 39% for 1 and 2 m SLR, respectively	49.12 km away from the entrance for base case – it moves upstream by 44% and 88% for 1 and 2 m SLR, respectively	13.25 km away from the entrance for base case – it moves upstream by 46% and 99% for 1 and 2 m SLR, respectively
	Tidal range pattern	X3	D1	D1	D1 but SLR takes cases to X3	D1	D1	D2	D1	D1
Medium (TR_0 = 1 m)	Location of minimum tidal range	37.75 km away from the entrance for base case – it moves upstream by 3% and downstream by 100% for 1 and 2 m SLR, respectively	25.55 km away from the entrance for base case – it moves upstream by 30% and 43% for 1 and 2 m SLR, respectively	6.80 km away from the entrance for base case – it moves upstream by 54% and 112% for 1 and 2 m SLR, respectively	65.37 km away from the entrance for base case – it moves upstream by 17% and downstream by 100% for 1 and 2 m SLR, respectively	30.38 km away from the entrance for base case – it moves upstream by 40% and 84% for 1 and 2 m SLR, respectively	8.00 km away from the entrance for base case – it moves upstream by 53% and 116% for 1 and 2 m SLR, respectively	79.12 km away from the entrance for base case – it moves upstream by 39% and 76% for 1 and 2 m SLR, respectively	33.25 km away from the entrance for base case – it moves upstream by 47% and 101% for 1 and 2 m SLR, respectively	8.50 km away from the entrance for base case – it moves upstream by 51% and 112% for 1 and 2 m SLR, respectively
	Tidal range pattern	D1 but SLR of 1m and 2m take cases to	D1	D1	D1 but SLR of 2m takes cases to X3	D1	D1	D1 but SLR of 2m takes cases to D2	D1	D1

		D2 and X3, respectively								
High (TR_0 = 4 m)	Location of minimum tidal range	22.30 km away from the entrance for base case – it moves upstream by 37% and 56% for 1 and 2 m SLR, respectively	11.35 km away from the entrance for base case – it moves upstream by 60% and 124% for 1 and 2 m SLR, respectively	2.40 km away from the entrance for base case – it moves upstream by 62% and 148% for 1 and 2 m SLR, respectively	37.12 km away from the entrance for base case – it moves upstream by 47% and 85% for 1 and 2 m SLR, respectively	14.75 km away from the entrance for base case – it moves upstream by 53% and 116% for 1 and 2 m SLR, respectively	3.62 km away from the entrance for base case – it moves upstream by 48% and 117% for 1 and 2 m SLR, respectively	39.25 km away from the entrance for base case – it moves upstream by 28% and 67% for 1 and 2 m SLR, respectively	14.75 km away from the entrance for base case – it moves upstream by 56% and 124% for 1 and 2 m SLR, respectively	3.50 km away from the entrance for base case – it moves upstream by 54% and 121% for 1 and 2 m SLR, respectively
	Tidal range pattern	D1	D1	D1	D1	D1	D1	D1	D1	D1
